# Effectiveness and safety of beta blockers in the management of hypertension in older adults: a systematic review to help reduce inappropriate prescribing

**DOI:** 10.1186/s12877-017-0575-4

**Published:** 2017-10-16

**Authors:** Anna Vögele, Tim Johansson, Anna Renom-Guiteras, David Reeves, Anja Rieckert, Lisa Schlender, Anne-Lisa Teichmann, Andreas Sönnichsen, Yolanda V Martinez

**Affiliations:** 1 South Tyrolean Academy of General Practice, via dei Vanga, Bolzano, Italy; 2 Institute of General Practice and Family Medicine, Paracelsus Medical University, Strubergasse, Salzburg, Austria; 3 Institute of General Practice and Family Medicine, Faculty of Health, University of Witten/Herdecke, Alfred-Herrhausen-Straße, Witten, Germany; 4 Department of Geriatrics, University Hospital Parc de Salut Mar, Passeig Marítim, Barcelona, Spain; 5 NIHR School for Primary Care Research, Manchester Academic Health Science Centre, Oxford Rd, Manchester, UK

**Keywords:** Systematic review, Beta blockers, Aged, Hypertension, Effectiveness, Inappropriate prescribing

## Abstract

**Background:**

The benefit from a blood pressure lowering therapy with beta blockers may not outweigh its risks, especially in older populations*.* The aim of this study was to look for evidence on risks and benefits of beta blockers in older adults and to use this evidence to develop recommendations for the electronic decision support tool of the PRIMA-eDS project.

**Methods:**

Systematic review of the literature using a stage approach with searches for systematic reviews and meta-analyses first, and individual studies only if the previous searches are inconclusive. The target population were older adults (≥65 years old) with hypertension. We included studies reporting on the effectiveness and/or safety of beta blockers on clinically relevant endpoints (e.g. mortality, cardiovascular events, and stroke) in the management of hypertension. The recommendations were developed according to the GRADE methodology.

**Results:**

Fifteen studies were included, comprising one meta-analysis, four randomized controlled trials, six secondary analyses of randomized controlled trials and four observational studies. Seven studies involved only older adults and eight studies reported subgroup analyses by age. With regard to a composite endpoint (death, stroke or myocardial infarction) beta blockers were associated with a higher risk of events then were other antihypertensive agents. Further, beta blockers showed no benefit compared to other antihypertensive agents or placebo regarding mortality. They appear to be less effective than other antihypertensive agents in reducing cardiovascular events. Contradictory results were found regarding the effect of beta blockers on stroke. None of the studies explored the effect on quality of life, hospitalisation, functional impairment/status, safety endpoints or renal failure.

**Conclusion:**

The quality of current evidence to interpret the benefits of beta blockers in hypertension is rather weak. It cannot be recommended to use beta blockers in older adults as first line agent for hypertension.

**Electronic supplementary material:**

The online version of this article (doi:10.1186/s12877-017-0575-4) contains supplementary material, which is available to authorized users.

## Background

Hypertension is an important public health challenge worldwide as nearly 1 billion people or ~26% of the adult population of the world suffers from hypertension [1, 2]. In Europe, the overall prevalence of hypertension appears to be around 30–45% of the general population [3], with a steep increase with ageing [4–6]. Hypertension is a major risk factor for cardiovascular and renal disease, and numerous clinical trials including studies in older adults have documented that effective treatment improves survival and confers cardiovascular benefits [4, 5]. The Framingham Heart Study and a meta-analysis done by Lewington et al. have shown a continuous and positive relationship between cardiovascular events and usual blood pressure above a baseline level of approximately 115/75 mmHg at all ages and in both sexes [6, 7]. Although the strength of the association is weakened with age [7], the absolute difference in cardiovascular risk between the highest and lowest usual blood pressure levels is much greater in older subjects [8]. Hence, it could be expected that especially older adults benefit from a blood pressure lowering therapy. However, there is a phenomenon called “reverse epidemiology” in older adults suffering from hypertension [9] which describes that higher blood pressure can be associated with increased survival [10]. The phenomenon likely reflects the confounding effects of other comorbid conditions that are quite common in this age group. This means that a lower blood pressure may be a proxy for poor health and a warning sign that further blood pressure lowering may actually be harmful. Thus, lower - for older adults - may not always be better [8]. Moreover, some studies suggest that in older adults tight blood pressure control (systolic blood pressure < 140 mmHg) may not reduce stroke more efficiently than a goal blood pressure < 150/90. In adults aged ≥75 years tight blood pressure may increase the incidence of cardiac and vascular disease, and renal failure [11, 12]. Tight blood pressure control is not recommended for patients with dementia or cognitive impairment or functional impairment and in patients with limited life expectancy (<2 years) [13].

Drugs used in treating hypertension are among the most commonly used drug classes that are associated with preventable drug-related hospital admissions especially in older populations [14, 15]. Among antihypertensive drugs, particularly beta blockers appear to be a problematical drug class for the treatment of hypertension [16]. A recently published Cochrane review on beta blockers for hypertension concludes that beta blockers are not recommended as first line treatment for hypertension as compared to placebo due to their modest effect on stroke and no significant reduction in mortality or coronary heart disease. Compared to other drug classes (diuretics, calcium channel blockers, and renin angiotensin system inhibitors) beta blockers were not efficient in decreasing mortality and morbidity [17]. It is argued that the use of beta blockers in older adults may not be justified because of physiologic changes in people over 60 years of age. These include a low cardiac output, bradycardia, high total peripheral resistance, reduced renal blood flow and glomerular filtration rate, and low plasma renin activity [18].

Nevertheless, to the best of our knowledge, so far no systematic review has analysed the specific evidence on the use of beta blockers for the management of hypertension in aged populations. This systematic review aims to explore the effectiveness and safety of beta blockers in the treatment of hypertension in older adults (≥65 years). A further aim was to develop recommendations on when to discontinue or to adjust the dose when using beta blockers in the treatment of hypertension in older adults. These recommendations were implemented in the electronic decision support tool of the PRIMA-eDS project (Polypharmacy in chronic diseases: Reduction of Inappropriate Medication and Adverse drug events in elderly populations by electronic Decision Support, www.prima-eds.eu).

## Methods

This systematic review was developed following an adaptation of the methods proposed by both the Cochrane Handbook for Systematic Reviews of Interventions [19] and the Preferred Reporting Items for Systematic Reviews and Meta-Analyses (PRISMA) [20]. A full description of the methods has been published previously [21]. A protocol for this systematic review has been elaborated and it is available from the authors on request.

### Study inclusion criteria

#### Types of studies

We included systematic reviews, meta-analyses, controlled interventional studies and observational studies reporting on risks and benefits of the use of beta blockers in the treatment of hypertension in older adults.

#### Types of participants

Older adults, ≥65 years old, with arterial hypertension were included. We ensured, that there was a sufficient number of older adults using the following criteria by type of design.

For systematic reviews and meta-analyses (any of the following criteria):

- overall mean or median age ≥ 65 years,
- overall mean or median age < 65, but subgroup analysis of participants <65, or
- overall mean or median age not reported but more than 80% of studies reported a mean or median age ≥ 65 years.

For controlled interventional studies and observational studies (any of the following criteria):

- ≥80% of participants ≥65 years or
- <80% of participants ≥65 years but subgroup analysis of participants <65. We included any setting reporting on the management of hypertension using beta blockers.

#### Types of interventions

We included studies reporting on the effectiveness and/or safety of beta blockers for the management of hypertension. Studies were included irrespective of beta blockers prescribed as monotherapy or in combination with any other drug for the treatment of hypertension. We included studies comparing beta blockers versus placebo, no treatment, other antihypertensive drugs or a non-pharmacological intervention.

#### Types of outcomes

We included any of the following clinically relevant endpoints as primary outcomes:

- Mortality
- Hospitalization
- Cardiovascular event including stroke
- Quality of life
- Adverse drug event
- Life expectancy
- Cognitive impairment or cognitive status
- Functional impairment or functional status
- Renal failure
- Composite end points including any of the above
- Any of the above evaluated as safety endpoints

### Study exclusion criteria

We excluded conference abstracts, pooled analyses, editorials, opinion papers, case reports, case series, narrative reviews, letters, and qualitative studies. We excluded studies evaluating only blood pressure values.

### Search method

Database searches were conducted by YVM and AW. We performed a stage approach with searches 1 and 2 in various databases for systematic reviews and meta-analyses, and search 3 for individual studies. During study selection for search 1 and 2, we identified eligible individual studies from excluded systematic reviews and meta-analyses and transferred those to search 3A for potential inclusion. The list of studies in search 3A was checked for inclusion following the procedures described in the section “Selection of studies”. Search 3B was designated as a last step to identify controlled interventional studies and observational trials in various databases. The respectively following search was performed only if the prior searches were inconclusive. We did not apply any language restriction to the search. The following paragraphs contain detailed information about search stages, databases and search dates:

- Search 1 was conducted on 11 September 2013 and updated on 22 December 2015 in the Cochrane Database of Systematic Reviews (OVID interface, 2005 to December 2015) and the Database of Abstracts or Reviews of Effects (DARE, OVID interface, 1991 to 2nd Quarter 2015).
- Search 2 was conducted on 17 October 2013 and updated on 22 December 2015 in MEDLINE (OVID interface, In-Process & Other Non-Indexed Citations 1946 to November Week 3 2015), EMBASE (OVID interface, 1974 to 2015 December 21), Health Technology Assessment (HTA, OVID interface 2001 to 4th Quarter 2015) and International Pharmaceutical Abstracts (IPA, OVID interface 1970 to December 2015).
- Search 3A consisted of eligible individual studies identified from systematic reviews and meta-analyses excluded from searches 1 and 2 covering the time of these searches.
- Search 3B was conducted on 28 September 2016 in MEDLINE (OVID interface, 2005 to 2016), EMBASE (OVID interface, 2005 to 2016), HTA (OVID interface 2005 to 2016) and IPA (OVID interface 2005 to September 2016). The result of search 3B was limited to the last 5 years (1 January 2011 to 28 September 2016) because search 3A had already used a high quality Cochrane review [17] which had covered all individual studies eligible for the time before 2012 (see 3A). This Cochrane review has recently been updated, and again, no additional eligible studies for our review were identified [22].
- Our hand search comprised a review of the bibliographies of all included studies.
- Study protocols from all searches were also collected to consider for future updates of this systematic review.

The PICOS-framework was used to develop the search strings. A selection of keywords used were: Population (older adults, aged) AND Condition (high blood pressure, hypertension) AND Intervention (beta blocker) AND Outcome (quality of life, mortality, life expectancy, hospitalization, cognitive impairment, functional impairment, cardiovascular event including stroke, renal failure) AND Study designs (systematic reviews, meta-analyses, controlled interventional studies and observational studies). Additional file 1 shows the full search strings. Endnote X7 was used to retrieve search results and to de-duplicate references.

### Selection of studies and data management

First, BF, AV and TJ assessed titles and abstracts from search 1 and 2 (including the search update) and identified studies to include using Endnote X7. Search 3A was done by YVM, TJ and AV, and search 3B by CS, AR, ALT, LS and BF. Second, full manuscripts were obtained for all titles and abstracts that appeared to meet the inclusion criteria or for cases of uncertainty for inclusion. ARG, YVM or AS were consulted when the two researchers could not reach an agreement on whether to include a study.

### Data extraction

Two reviewers (AV, YVM or TJ respectively CS, AR, ALT, LS, SW or BF) independently conducted data extraction of the included studies using a standardised and piloted data collection form which was published with the protocol [23]. The two reviewers checked each other’s data extraction to look for completeness and accuracy. The data collection form included information related to the study design and aim, characteristics of the participants (i.e. age, sex, setting, comorbidity, use of concomitant medications, frailty, functional and cognitive status), the intervention (i.e. beta blockers), comparison, time to follow-up, and reported outcomes.

### Quality appraisal

We used three validated assessment tools to evaluate the quality of each study design: for systematic reviews/meta-analyses the measurement tool to assess systematic reviews (AMSTAR) [24, 25] for intervention studies the Cochrane Collaboration’s tool for assessing risk of bias [26] and for observational studies the Critical Appraisal Skills Programme (CASP) [27, 28]. Risk of bias was assessed independently by at least two review authors according to the Cochrane Handbook [26].

### Data synthesis

Methods utilised to synthesise the studies depended on their quality, design and heterogeneity. According to the protocol we wanted to pool results of studies if at least two were homogeneous regarding participants, interventions and outcomes [23]. If that was not possible due to differences in the reporting or substantive heterogeneity, we reported a narrative synthesis describing all included studies, participants and findings. No additional meta-analyses were performed.

### Identification of additional “references of interest” and the development of recommendations

During the search process, BF, AV, TJ, YVM and ARG identified additional references for the development of recommendations according to the methodology described by Martinez-Renom Guiteras et al. [21]. We identified nine additional references which can be seen in Additional file 2. Two were found in search 2, one in the search update, one in the hand search of references of included studies, two were suggested by two clinical researchers (IK and MMV) and three were found by snowballing [29]. The studies did not meet our inclusion criteria (mainly due to age < 65 years), but provided supportive information for the formulation of the recommendations. Included studies and additional references were summarised in a document that was used in team meetings to discuss recommendations on when the use of beta blockers should be discontinued or new doses should be considered in older adults for the management of hypertension [21]. Each recommendation was given strength (weak or strong) and quality (low, moderate or high) following the GRADE methodology [30–32]. The recommendations are provided in Table 1.

**Table 1:** Recommendations for beta blockers in older people with hypertension

Recommendation	Strength of the recommendation	Quality of the evidence	Type of evidence
It is suggested to discontinue the beta blocker or change it to another antihypertensive drug (unless another indication for beta blockers exists), because beta blockers may increase the risk of stroke and other composite cardiovascular outcomes compared to other antihypertensive agents while not revealing any benefit regarding cardiovascular outcomes or mortality compared to placebo for adults >60 years.	Strong	Low Downgraded for indirectness because only one meta-analysis and one RCT were focused on older people	2 Cochrane reviews [17, 49], 2 meta-analyses [33, 54], 2 recommendation papers from the Canadian Hypertension Education Program [51, 52], and 1 RCT [35]
It is suggested to discontinue atenolol for the management of hypertension because it appears to be less effective than other antihypertensives in reducing cardiovascular events, and to have a higher risk of adverse events.	Strong	Low Downgraded for indirectness because only one RCT was focused on older people	2 Cochrane reviews [17, 50] and 2 RCTs [35]
It is suggested to discontinue beta blockers as monotherapy for the management of hypertension because it may be inferior to other antihypertensives in preventing stroke, and not to have any benefits in decreasing the rates of cardiovascular events. This recommendation does not apply if the patient has other indications for beta blockers (heart failure, arrhythmia, previous myocardial infarction, angina pectoris).	Strong	Low Downgraded for indirectness because only the meta-analysis included a subgroup analysis in older people	1 Cochrane review [17], 1 meta-analysis [33] and 1 evidence based guideline [38]

Legend: *RCT* randomized controlled trial

## Results

### Results of the search

**Fig. 1:**
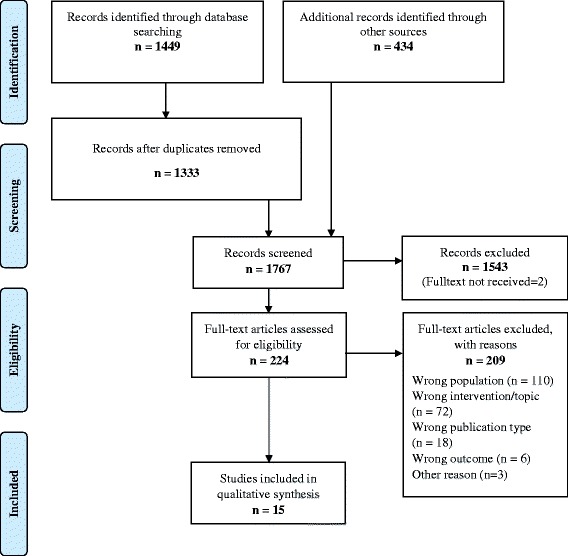
Preferred Reporting Items for Systematic Reviews and Meta-Analyses (PRISMA) flow diagram

We identified 1449 records through database searching and 434 through other sources (hand search of reference lists of included studies). After removing 116 duplicates, we screened 1767 records and excluded 1543 records checking titles and abstracts. We assessed 224 full texts for eligibility and excluded 209 records. Additional file 3 provides the comprehensive list of reasons for exclusion of studies after full text analysis. The PRISMA flow diagram is presented in Fig. 1.

### Characteristics of the included studies

We included one meta-analysis [33], four randomized controlled trials [34–37], six secondary analyses (post hoc analyses) of randomized controlled trials [36, 38–42], and four observational studies [43–46] (three cohort studies and one cross sectional study). Table 2 shows details of the included meta-analysis, and Table 3 shows details of the included trials. Follow-up in the included trials ranged from 9 months [36] to a mean of 5.8 years [35]. Six studies were carried out in the UK [35–39, 42], two in Japan [41, 47] and single studies in Italy [46], Sweden [43], and the USA [45]. Two studies were multicentre trials conducted in European countries [40, 44], and one study was conducted in 14 countries worldwide [34].

**Table 2:** Data extraction of the meta-analysis

Khan et al. 2006. Re-examining the efficacy of β–blockers for the treatment of hypertension: a meta-analysis, CMAJ, 174(12): 1737-42 [33]		
Country	Canada	
Funding	Not stated	
Setting	Not stated	
Objective	To explore the efficacy (stroke, myocardial infarction and death) of beta blockers in different age groups	
**Inclusion and exclusion criteria**		
Population	Hypertension, distinguish between “younger” patients <60 years (mean age ranged from 45.5 to 56.2 years, n=10 trials and n=50,612 patients) and “older” patients ≥60 years (60.4 to 76 years, n=11 trials and n=95,199 patients, 9 trials out of 11 with mean age ≥65 years).	
Intervention	Beta blocker as first-line therapy for hypertension in preventing major cardiovascular events	
Control	No treatment, placebo, diuretic, ACE inhibitor, calcium-channel blocker, angiotensin-receptor blocker	
Outcomes	Stroke, myocardial infarction, or death	
Study designs	Randomized controlled trials	
**Methods**		
Study design	Systematic review including meta-analysis. Results of the individual studies are combined to produce an overall statistic.	
Last date searched	18 January 2006	
Data bases searched	PubMed (1950-18.01.2006)	
Other sources searched	Hand search, reference lists of published hypertension meta-analysis (MEDLINE) and the Cochrane Library. Contacted Canadian hypertension experts.	
Number of included studies	21 Randomized controlled trials	
Number of included patients	145,811	
**Outcomes, results**		
**Primary**		
Composite cardiovascular outcome of death, nonfatal myocardial infarction or nonfatal stroke	Beta blockers reduced event rates compared with placebo (RR 0.86, 95% CI 0.74–0.99, based on 794 events in 19,414 patients), in trials enrolling younger patients, but benefits were not found in trials enrolling older patients (RR 0.89, 95% CI 0.75–1.05, based on 1115 events in 8,019 patients). Among 30,412 patients younger than 60 years of age, there was no difference in event rates between those randomly assigned to beta blockers therapy compared with those receiving other antihypertensive agents (1515 events, RR 0.97, 95% CI 0.88–1.07). However, in the 79,775 patients 60 years of age or older, beta blockers were associated with a higher risk of events than other antihypertensive agents (7405 events, RR 1.06, 95% CI 1.01–1.10).	
**Secondary**		
**Beta blocker compared to placebo**	**“Younger population” < 60 years**	**“Older population” ≥ 60 years**
Death	RR 0.94, 95% CI 0.79–1.10	RR 0.91, 95% CI 0.74–1.12
Nonfatal myocardial infarction	RR 0.85, 95% CI 0.71–1.03	RR 0.98, 95% CI 0.83–1.16
Nonfatal stroke	RR 0.84, 95% CI 0.65–1.10	RR 0.78, 95% CI 0.63–0.98
Heart failure	RR 1.05, 95% CI 0.72–1.54	RR 0.54, 95% CI 0.37–0.81
**Beta blocker compared to other antihypertensive agents**	**“Younger population” < 60 years**	**“Older population” ≥ 60 years**
Death	RR 0.97, 95% CI 0.83–1.14	RR 1.05, 95% CI 0.99–1.11
Nonfatal myocardial infarction	RR 0.97, 95% CI 0.86–1.10	RR 1.06, 95% CI 0.94–1.20
Nonfatal stroke	RR 0.99, 95% CI 0.67–1.44	RR 1.18, 95% CI 1.07–1.30
Heart failure	RR 0.93, 95% CI 0.64–1.34	RR 0.98, 95% CI 0.87–1.11
**Subgroup analysis, ≥60 years**		
**Beta blocker compared to placebo or no treatment** (5 trials and n=8,019 patients, range mean age 65 to 75.7 years)		
Composite cardiovascular outcome of death, nonfatal myocardial infarction or nonfatal stroke	Beta-blockers´ benefits were not found in trials enrolling older patients RR 0.89, 95% CI 0.75–1.05, based on 1,115 events in 8,019 patients.	
Death	RR 0.98, 95% CI 0.83–1.16	
Nonfatal myocardial infarction	RR 0.98, 95% CI 0.83–1.16	
Nonfatal stroke	RR 0.78, 95% CI 0.63–0.98	
Heart failure	RR 0.54, 95% CI 0.37–0.81	
**Beta blocker compared to other antihypertensive agents** (7 trials and n=87,180 patients, range mean age 60.4 to 76 years)		
Composite cardiovascular outcome of death, nonfatal myocardial infarction or nonfatal stroke	Beta blockers were associated with a higher risk of events than were other antihypertensive agents (7,405 events, RR 1.06, 95% CI 1.01–1.10).	
Death	RR 1.05, 95% CI 0.99–1.11	
Nonfatal myocardial infarction	RR 1.06, 95% CI 0.94–1.20	
Nonfatal stroke	RR 1.18, 95% CI 1.07–1.30	
Heart failure	RR 0.98, 95% CI 0.87–1.11	
**Conclusion**		
Beta blockers should not be considered first-line therapy for older hypertensive patients without another indication for these agents; however, in younger patients beta blockers are associated with a significant reduction in cardiovascular morbidity and mortality.		
**Quality appraisal**		
**Quality criteria for systematic reviews and meta-analyses**	**Author´s judgement**	**Support for judgement**
Precise and accurately defined research question (e.g. PICO)	Yes	Criteria for considering studies are explicitly explained in the paper. The PICOS scheme can be applied
Well-defined selection criteria	Yes	See above
Was an ‘a priori’ design provided?	No	There is no published protocol for this meta-analysis
Systematic literature research	Yes	Search method is illustrated
Appropriate search strings, data bases and hand search	No	Only a PubMed search and a hand search were conducted. A very limited search string was used.
At least two reviewers for selecting retrieved studies	Unclear	The review process, screening abstracts and reading full texts is not described. Two authors extracted outcome data from each trial independently. The inter observer kappa for trial inclusion was 0.94
Well documented process of selection of included studies (e.g. PRISMA flow diagram)	Unclear	Some kind of PRISMA flow was used, but the review process is unclear. A list of excluded studies is missing.
Quality of the studies documented and considered for the synthesis of evidence	No	Quality appraisal of studies is lacking
Was the conflict of interest stated?	Yes	None declared.
Assessed publication bias	No	Publication bias not assessed
Heterogeneity statistically analysed	Yes	X^2^ tests were used to test heterogeneity
**Quality of internal validity**	Poor	No study quality assessment was performed

*Legend: RCT* randomized controlled trial, *ACE* angiotensin-converting enzyme, *ARB* angiotensin-receptor blockers, *BB* Beta-blockers, *CCB* calcium channel blockers, *FU* Follow up, *TD* Thiazide diuretic

**Table 3:** Summary of characteristics of the included studies

Author year	Type of study	Aim	Intervention/control	Sample size	Follow-up	Outcomes and measurement tools if applicable
Carlsson 2014 [43]	Cohort study	To study mortality rates in men and woman with hypertension and AF prescribes different cardiovascular pharmacotherapies (time to death between registration of AF diagnosis and 31.12.2007)	Antithrombotic drugs alone: - antiplatelet agents vs. no treatment - anticoagulants vs. no treatment - anticoagulants vs. antiplatelets Beta blocker: - Selective vs. no treatment - non-elective vs. no treatment - non-selective vs. selective RAS-blocking agents: - vs. no treatment - ARBs vs. ACE inhibitors - RAS blocking agents + other vs. no treatment - ARB + thiazide vs. ACE inhibitor + thiazide Calcium receptor- blocking agents: - Vessel selective vs. no treatment - Heart active vs. no treatment - Vessel selective vs. heart active - - Statins	*n* = 5602 4748 aged ≥65 y 854 aged <65 y	Mean follow-up 3.4 years	Mortality
Collier 2011 [42]	Multicentre, international randomized trial	To evaluate the efficacy and safety of an amlodipine versus an atenolol-based antihypertensive regimen among older (≥ 65 years) and younger (<65 years) patients.	Atenolol-based (atenolol ± thiazide diuretic) Amlodipine-based (amlodipine ± perindopril)	*n* = 19,257 8137 aged ≥65 and 11,020 aged <65	Median follow up 5.5 years	Nonfatal myocardial infarction and fatal coronary heart disease and cardiovascular events
Coope 1986 [34]	RCT	To explore, if the treatment of hypertension in patients between the ages of 60 and 79 years old reduces the incidence of stroke or coronary disease or affects cardiovascular or overall mortality	Intervention group: Step 1: atenolol 100 mg If blood pressure control was insufficient the further treatment steps were applied: Step 2: bendrofluazide 5 mg Step 3: alphamethyldopa 500 mg (and eventually nifedipine retard 20 mg or other antihypertensive medication) Control group: usual care	*n* = 884, intervention group *n* = 419 control group *n* = 465	Mean follow up 4.4 years (range 1–10, SD not reported)	*Primary outcomes*: myocardial infarction, major strokes, minor strokes, transient ischaemic attacks, death *Secondary outcomes*: Congestive heart failure Heart failure Atrial fibrillation Clinical gout Diabetes Non- fatal cancer Vertigo Dizzy spells
Subgroup analysis in Coope 1986 [34]	See above	To analyse whether the treatment of hypertension in patients between the ages of 70 and 79 years old reduces the incidence of all strokes	See above	Not stated	Not stated	Incidence of all strokes including major strokes, minor strokes, transient ischaemic attacks
Gelber 2013 [45]	Prospective, community- based cohort study	To determine the associations between classes of antihypertensive medication use and the risk of cognitive impairment among elderly hypertensive men.	No drug, BB alone, ACE alone, Diuretic alone, CCB alone, vasodilators alone, BB & 1 other, Other drug combinations	*n* = 2197	Median follow up 5.8 years (SD 5.1)	Development of cognitive impairment CASI Score, a combination of the Hasegawa Dementia Screening Scale, the Folstein Mini-Mental State Examination, and the Modified Mini-Mental State Examination
Pepine 2003 [32]	RCT	To test the hypothesis, that the risk for adverse outcomes in older people with hypertension and coronary artery disease treated with a calcium antagonist based strategy or a non-calcium antagonist (atenolol) based strategy is equivalent.	*Step 1:* either calcium antagonist group: 240 mg/d of verapamil sustained release or non-calcium antagonist: 50 mg/d of atenolol If target blood pressure was not achieved, further steps: *Step 2:* calcium antagonist group additional trandolapril non-calcium antagonist group additional hydrochlorothiazide *Step 3*: dosage increase *Step 4:* calcium antagonist group additional hydrochlorothiazide non-calcium antagonist group additional trandolapril.	*n* = 22,576 Calcium antagonist group, *n* = 11,267 Non-calcium antagonist group, *n* = 11,309	Mean follow up 2.7 years (range 1 day to 5.4 years, SD not reported)	*Primary outcome:* first occurrence of -death (all cause) -non-fatal myocardial infarction -non-fatal stroke *Secondary outcomes:* death (all cause), non-fatal myocardial infarction, non-fatal stroke, time to most serious event (ranked from death as most serious, to myocardial infarction, to stroke as last serious), cardiovascular death, angina, cardiovascular hospitalizations, cancer, Alzheimer disease, Parkinson, gastrointestinal bleeding, blood pressure control, new diagnosis of diabetes, adverse experiences.
Subgroup analysis in Pepine 2003 [32]	See above	See above	See above	Subgroup >70 years old: calcium antagonist group: *n* = 3694 non- calcium antagonist group: *n* = 3829	See above	See above
Ruwald 2012 [41]	Post hoc analysis of a double-blind, double-dummy randomized trial	To investigate the influence of age on the effects of losartan versus atenolol-based antihypertensive treatment	Intervention: losartan 50 mg (step 2: + HCT 12.5 mg HCT step 3: losartan 100 mg + HCT 12.5 mg step 4: losartan 100 mg + HCT 12.5-25 mg HCT + another antihypertensive treatment) Control: atenolol 50 mg (step 2: + HCT 12.5 mg step 3: atenolol 100 mg + HCT 12.5 mg step 4: atenolol 100 mg + HCT 12.5-25 mg + another antihypertensive treatment)	*n* = 9193 *n* = 4304 < 67 years (46.8%) *n* = 4889 ≥ 67 years (53.2%)	Mean follow up 4.8 years	*Primary outcome:* Composite of cardiovascular death, nonfatal stroke, nonfatal MI
Testa 2014 [44]	Cross sectional study	To evaluate long-term mortality in hypertensive older adults taking atenolol	No atenolol (Intervention)/atenolol (control)	972 patients aged ≥65 with isolated hypertension mean age 74.4 ± 6.4	Follow up of 12 years from 1992 to 2003 (not stated if median or mean follow up)	Taking atenolol showed a greater mortality (73.9% vs 55.0%; *p* = 0.047) and higher pulse arterial pressure values (74.7 vs 63.02 mmHg, *p* < 0.001) than those not taking atenolol. Atenolol use (HR 1.91; 95% CI: 1.04–4.31; *p* = 0.04) was predictive of long-term mortality. Pulse arterial pressure (HR 1.02; 95% CI 1.01–1.03; *p* = 0.032) was weakly predictive of long-term mortality.
**Studies based on the COPE (Combination Therapy of Hypertension to Prevent Cardiovascular Events) trial**						
Matsuzaki 2011 [35] Main trial	multicentre, prospective, randomized, open-label, blinded-endpoint trial	To determine the optimal CCB benidipine-based combination therapy (angiotensin-receptor blocker (ARB), beta-blocker or a thiazide) in terms of lowering BP and preventing cardiovascular events	3 intervention arms: 1. ARB (benidipine 4 mg + angiotensin receptor blocker usual dose) 2. BB (benidipine 4 mg + beta-blocker usual dose) 3. TD (benidipine 4 mg + half daily dose of thiazid diuretic step 2: benidipine 8 mg (plus ARB/BB/TD) step 3:benidipine 8 mg plus dose increase of ARB/BB/TD	*n* = 3293 1. ARB *n* = 1110 (analysed) 2. BB *n* = 1089 (analysed) 3. TD *n* = 1094 (analysed) *n* = 1533 ≥ 65 years (46.6%) *n* = 1760 < 65 years (53.4%)	Median follow up 3.61 years	*Primary outcome*: composite of cardiovascular morbidity and mortality (sudden death, fatal or non-fatal stroke, fatal or non-fatal myocardial infarction, hospitalization due to unstable angina, new onset of heart failure new onset or worsening of peripheral arterial disease, new onset or worsening of renal failure) *Secondary outcomes:* (1) All-cause mortality (2) Death from cardiovascular events (3) Fatal and non-fatal cardiovascular events (4) New onset of diabetes mellitus (5) Safety (adverse events and adverse drug reaction) (6) Nonfatal stroke Hospitalization due to heart failure (results on outcome not reported)
Ogihara 2012 [40] COPE trial	post hoc analysis of the COPE trial	To evaluate the efficacy and safety in older (≥65 years) and younger (<65 years) hypertensive patients				
**Studies based on the Medical Research Council (MRC) trial**						
MRC 1992 [33] Main trial	RCT	To establish whether treatment with diuretic or beta blocker in hypertensive older adults reduces risk of stroke, coronary heart disease, and death.	Patients were randomized to atenolol 50 mg daily; hydrochlorothiazide 25 mg or 50 mg plus amiloride 2.5 mg or 5 mg (Moduretic©) daily; or placebo.	*n* = 4396 Three groups (a) potassium sparing diuretic regimen (hydrochlorothiazide +amiloride) *n* = 1081 (b) beta blocker atenolol *n* = 1102 (c, d) matching placebo tablets *n* = 2213	Mean follow up 5.8 years 25,355 patients years of observation (SD not reported)	Strokes, coronary events, and deaths from all causes.
Bird 1990 [36]	RCT, secondary analysis	To explore if cognitive dysfunction, manifested as an increased incidence of confusional states or impaired concentration and attention are associated either with a particular antihypertensive regime or with the reduction in blood pressure level that it induces. To explore if a continuous reduction in mildly elevated blood pressure levels affect the rate of change in cognitive function.	Patients were randomly assigned to hydrochlorothiazide 25 mg and 2.5 mg amiloride, (Moduretic©) daily, atenolol 50 mg daily, or placebo.	*n* = 2401 hydrochlorothiazide amiloride 24% atenolol 24% Placebo 52%	Results reported at month 1 and 9 (median or mean follow up and SD not reported)	Cognitive tests: Verbal intelligence measured with the Nelson Adult Reading Test (NART), Non verbal intelligence (performance ability) measured with the Ravens Matrice, part a & b, Capacity to learn and remember new material measured with the Paired Associate Learning Test (PALT), Alertness and speed of reaction measured with the Trail-Making Test (TMT), Depression with the self-rating Depression Questionnaire (DQ) Blood Pressure
Carr 2012 [37]	RCT, secondary analysis	To assess the impact of the blood pressure profile on cardiovascular risk in the Medical Research Council (UK) elderly trial and to investigate whether the effects of hypertensive drugs in reducing event rates are solely a product of systolic pressure reduction.	Intervention:1. atenolol 50 mg daily or 2. hydrochlorothiazide 25 mg (50 mg) + amiloride 2,5 mg (5 mg) Control: Placebo	*n* = 4396 atenolol: *n* = 1102 hydrochlorothiazide + amiloride: *n* = 1081 hydrochlorothiazide + Placebo *n* = 2213	Mean follow up 5.8 years	*Primary outcomes:* stroke CHD *secondary outcomes:* association between BP and outcomes
Lever 1992 [48]	RCT, secondary analysis	To study the effect of two treatments for hypertension on all-cause mortality and morbidity from cardiovascular disease	see above	see above	see above	Stroke coronary events all cardiovascular events all-cause mortality withdrawal/loss to FU from treatment
Lever 1993 [38]	RCT, secondary analysis	To determine whether hypotensive drug treatment in men and women aged 65–74 reduces stroke, CHD and mortality	see above	see above	see above	Stroke coronary events all cardiovascular events all-cause mortality change in blood pressure

*Legend: RCT* randomized controlled trial, *ACE* angiotensin-converting enzyme, *ARB* angiotensin-receptor blockers, *BB* Beta-blockers, *CCB* calcium channel blockers, *FU* Follow up, *TD* Thiazide diuretic

### Participants

Table 4 shows the characteristics of the participants in the included studies. Nine studies analysed older adults (age ≥ 65 years) [35, 36, 39, 42–46, 48] and another fife studies included a subgroup analysis of patients 65 years or older [34, 37, 40, 41, 47]. The meta-analysis performed a comparison between studies enrolling patients <60 years vs. those enrolling patients ≥60 years in a subgroup analysis [33]. The studies included in this group of studies enrolling patients ≥60 years met our inclusion criteria as nine out of the 11 reported a mean age of participants of ≥65 years (82% of the studies with mean age ≥ 65 years) [33]. Male sex ranged from 29% [49] to 100% [45]. Two trials reported ethnicities [34, 45]. Comorbidity was reported in all studies except in the MRC trial [35]. Six studies reported on use of concomitant medications [34, 41, 43, 44, 46, 47]. Frailty and/or cognitive status were only reported in two studies [45, 46].

**Table 4:** Characteristics of the participants in the included studies

Author year	Setting / country / ethnicity	Male sex	Age^a^	Reported comorbidities	Reported concomitant medications	Functional status/Frailty level	Cognitive status
Carlsson 2014 [43]	Sweden	50.14%	Men: mean age 72.3 years, 79% ≥ 65 years. Woman: mean age 77.2 years, 92% ≥ 65 years	Diagnosis of both AF and hypertension (inclusion criteria), coronary heart disease, cerebrovascular diseases, including intracranial bleeding and peripheral embolism, congestive heart failure, DM II	- Diuretic drugs (thiazides, related agents, combined formulations with other drugs, loop diuretics, potassium-saving diuretics) - Beta blocker (ß1-selective, non-selective) - Calcium receptor-blocking agents (vessel-selective, heart-selective) - RAS-Blockers (ACE-inhibitors, ARBs) - Lipid-lowering drugs (statins) - antithrombotic drugs	Not reported	Not reported
Collier 2011 [44]	UK, Sweden, Denmark, Iceland, Norway, and Finland	Patients ≥65 Amlodipine-based regimen 73.6% Atenolol-based regimen 73.5%	Patients ≥65 Amlodipine-based regimen 71.1 (4.0) Atenolol-based regimen 71.1 (4.0)	Type 2 diabetes mellitus, ECG abnormalities (not LVH), LVH (on ECG or ECHO), Peripheral vascular disease	No previous antihypertensive use, n (%): Amlodipine-based regimen 681 (16.8) Atenolol-based regimen 677 (16.5) Aspirin use, n (%): Amlodipine-based regimen 1066 (26.4) Atenolol-based regimen 1046 (25.5)	Not reported	Not reported
Coope 1986 [37]	13 general practices in England and Wales	Intervention group: 29.0% Control group: 32.0%	Intervention group: 68.7 (5.2) years Control group: 68.8 (5.1) years	Intervention group Left ventricular hypertrophy on ECG: *n* = 8 Cardiac enlargement on chest XRay: *n* = 22 Control group Left ventricular hypertrophy on ECG: *n* = 11 Cardiac enlargement on chest XRay: *n* = 21	Not stated	Not stated	Not stated
Subgroup analysis in Coope 1986 [37]	See above	29.1% (based on own calculations)	70–79 years	Not stated	See above	See above	See above
Gelber 2013 [45]	Hawaii, USA (Japanese men)	100%	Overall mean age: 77 No drug 77.2 (4.2) BB alone 76.2 (3.8) ACE alone 76.7 (4.1) Diuretic alone 77.4 (4.0) CCB alone 76.9 (4.0) Vasodilators alone 76.8 (3.6) BB & 1 other 76.5 (3.6) Other drug combi-nation 77.0 (4.0)	Type 2 diabetes mellitus, CVD (defined as history of myocardial infarction, angina and other coronary heart disease or stroke), APOE	Not reported	Not reported	BB use was not significantly associated with a lower risk of cognitive impairment, suggesting that medication class may be less relevant if the SBP is not adequately controlled.
Pepine 2003 [34]	862 primary care sites in 14 countries worldwide Calcium antagonist group (%): White 48.5 Hispanic 35.7 Black 13.4 Asian 0.6 Other/multiracial 1.9 Non-calcium antagonist group (%): White 48.3 Hispanic 35.6 Black 35.6 Asian 0.8 Other/multiracial 1.9	Calcium antagonist group (%): 48.1 Non-calcium antagonist group (%): 47.7	Calcium antagonist group: 66,0 (9.7) years Non-calcium antagonist group: 66,1 (9.8) years	Calcium antagonist group (%): Myocardial infarction 32.1 Prior myocardial infarction or abnormal angiogram 52.6 Angina pectoris 66.2 CABG ≥1 month ago 15.5 PCI ≥ 1 month ago 15.2 CABG or PCI 27.3 Stroke 5.3 Left ventricular hypertrophy 21.5 Unstable angina ≥1 month ago 11.4 Arrhythmia 7.1 Heart failure class II-III 5.5 Peripheral vascular disease 11.9 Diabetes 28.1 Hypercholesterolemia 55.9 Renal impairment 1.9 Cancer 3.5 Non-calcium antagonist group (%): Myocardial infarction 31.8 Prior myocardial infarction or abnormal angiogram 53.3 Angina pectoris 67 CABG ≥1 month ago 16.1 PCI ≥ 1 month ago 14.7 CABG or PCI 27.3 Stroke 5.0 Left ventricular hypertrophy 22.3 Unstable angina ≥1 month ago 11.5 Arrhythmia 7.1	Calcium antagonist group (%): Aspirin© or other antiplatelet inhibitors 57 NSAIDs^b^ 17.6 Antidiabetic medication 22.1 Any lipid-lowering agent 36.8 Nitrates 35.4 Potassium supplement 6.9 Hormone replacement 17.7 Non-calcium antagonist group: Aspirin© or other antiplatelet inhibitors: 56.4 NSAIDs: 17.9 Antidiabetic medication: 22.9 Any lipid-lowering agent: 36.6 Nitrates: 36.6 Potassium supplement 6.9 Hormone replacement 18.5	Not stated	Not stated
				Heart failure class II-III 5.6 Peripheral vascular disease 12.0 Diabetes 28.6 Hypercholesterolemia 55.6 Renal impairment 1.9 Cancer 3.3			
Subgroup analysis in Pepine 2003 [34]	Not stated	Not stated	>70 years	Not stated	Not stated	Not stated	Not stated
Ruwald 2012 [40]	Denmark, Finland, Iceland, Norway, Sweden, and UK,	45–66 years: losartan: 51.2% atenolol: 50.6% 67–83 years: losartan: 41.3% atenolol: 42.0%	overall mean age 67 years 46.8% <66 years 53.2% ≥67 years	Any vascular disease: 25% (Coronary heart disease 16%, cerebrovascular disease 8%, peripheral vascular disease 6%), atrial fibrillation: 4% diabetes: 13% isolated systolic hypertension: 14%	Not reported	Not reported	Not reported
Testa 2014 [46]	Campania/Southern Italy	41%	Mean age 74.4 (±6.4) 93% ≥ 65 y.	Hypertension (inclusion criteria) Diabetes, chronic renal failure, AF	ACE-inhibitors, diuretics, hypolipidemic drugs	BADL (basic activities of daily living) GDS (geriatric depression scale)	MMSE: Atenolol vs. no Atenolol 24.9 vs. 25.1 (*p* = 0.841)
**Studies based on the COPE (Combination Therapy of Hypertension to Prevent Cardiovascular Events) trial**							
**Author year**	**Setting / country / ethnicity**	**Male sex**	**Age**	**Reported comorbidities**	**Reported concomitant medications**	**Functional status**/**Frailty level**	**Cognitive status**
COPE trial Matsuzaki [47] Main trial	Japan	Overall: benidipine plus -ARB: 51% -BB: 50.5% -TD: 50.5%	Benidipine plus -ARB: 63.0 (10.6) -BB: 63.2 (10.8) -TD: 63.1 (10.8)	ARB/BB/TD groups: Overall: previous casdiovascular disease: 13%/11.4%/12.5% Arrhythmia: 2.7%/3.0%/2.4% Diabetes: 13.9%/14.2%/14.4%	ARB/BB/TD groups: Overall: Antiplatelet agents: 8.9%/6.8%/7.3% Lipid-lowering agents: 21.1%/20.4%/21.2% Antidiabetic agents: 6.9%/7.3%/7.2%	Not reported	Not reported
Ogihara [41]		≥65 years: -ARB: 43.6% -BB: 43.8% -TD: 42.3%	46.6% aged ≥65 years (≥65 years: mean age 72.6 years)	≥65 years: previous cardiovascular disease: 18.3% stroke: 4.4% angina pectoris: 4.8% MI: 1.05/0.7%/1.2% Arrhythmia: 3.6%/3.9%/3.6% Diabetes: 16.5%/16.5%/16.8%	≥65 years: Antiplatelet agents: 13.7%/10.7%/11.8% Lipid-lowering agents: 23.1%/21.7%/20.4% Antidiabetic agents: 8.6%/9.2%/9.0%		
**Studies based on the Medical Research Council (MRC) trial**							
**Author year**	**Setting / country / ethnicity**	**Male sex**	**Age**	**Reported comorbidities**	**Reported concomitant medications**	**Functional status**/**Frailty level**	**Cognitive status**
MRC 1992 [35] Main trial	226 general practices in England, Scotland, and Wales	Diuretic 42.0% Beta blocker 41,0% Placebo 42,0%	Range 65–74 years.	Not stated	Not stated	Not stated	Not stated
Bird 1990 [36]	see above	41.0%	Mean (SD) 70.3 (2.7) years	see above	see above	see above	see above
Carr 2012 [42]	see above	42%	Mean age 70.3 years Placebo 70.3 Diuretic 70.3 b-blocker 70.4	see above	see above	see above	see above
Lever 1992 [48]	see above	see above	see above	see above	see above	see above	see above
Lever 1993 [39]	see above	see above	see above	see above	see above	see above	see above

*Legend: CABG* coronary artery bypass graft, *PCI* percutaneous coronary interventions, *SD* standard deviation, ^a^ Mean age (SD) years, ^b^ Non Steroid Anti Inflammatory Drugs, *APOE* Apolipoprotein E, *ARB* angiotensin-receptor blocker, *BB* beta-blocker, *CVD* cardiovascular disease, *MI* Myocardial infarction, *GDS* Geriatric Depression Scale, *TD*: thiazide diuretic

### Interventions and outcomes

Detailed information about interventions and outcomes can be found in Table 3: Summary of characteristics of the included studies.

### Any beta blocker

The meta-analysis [33] included only randomized controlled trials evaluating the efficacy of beta blockers as first-line therapy for hypertension in preventing major cardiovascular events (i.e. stroke, myocardial infarction or death). In the analyses of the group of studies that we are including, the authors compared beta blockers to no treatment, placebo, diuretics, ACE-inhibitors, calcium-channel blockers and angiotensin-receptor blockers. The Matsuzaki trial [47] and the related secondary analysis of Ogihara et al. [41] analysed the optimal calcium-channel blocker benidipine-based combination therapy comparing three intervention arms, one with additional angiotensin receptor blocker, one with a beta blocker and one with a thiazide diuretic. The primary outcomes of these studies were the composite of cardiovascular morbidity and mortality. The observational study of Carlsson et al. [43] compared the following beta blocker treatments: selective vs. no treatment, non-selective no treatment and non-selective vs selective with regard to the outcome of mortality. Gelber et al. [45], another observational study, analysed beta blocker alone and beta blocker in combination with one other antihypertensive drug for the outcome of the development of cognitive impairment.

### Atenolol

Atenolol (50 or 100 mg/d) was examined in three randomized controlled trials [34, 35] and the related secondary analyses [36, 38, 39, 42], a post hoc analyses of a randomized controlled trial [40] and in two observational studies [44, 46]. In Pepine et al. [34] atenolol was compared to a treatment with verapamil, in the MRC trial [35] and its related secondary analyses it was compared with hydrochlorothiazide plus amiloride (Moduretic©) or placebo [36, 38, 39, 42], and in Coope et al. [34] atenolol was compared to no treatment. In Ruwald et al. [40], atenolol was compared to losartan plus hydrochlorothiazide and in the observational study of Collier et al. [44], an atenolol based treatment was compared with an amlodipine based treatment. The observational study of Testa et al. [46] compared atenolol to no- atenolol. The primary outcomes of these trials can be seen in Table 3.

### Main findings

The results of the included studies are displayed in Tables 5 and 6 and summarized below.

**Table 5:** Summary of study findings of the meta-analysis (subgroup analysis)

Author year	Objective
Kahn 2006 [33]	To explore the efficacy (stroke, myocardial infarction and death) of beta blockers in “younger”(< 60 years) and “older” (≥ 60 years) patients.
**Results:**	
**1. Beta blockers compared to placebo or no treatment** ^**1**^ (5 trials and *n* = 8019 patients, range mean age 65 to 75.7 years)	
**Primary outcome:** composite cardiovascular outcome of death, nonfatal myocardial infarction or nonfatal stroke	Beta blockers´ benefits were not found in trials enrolling older patients RR 0.89, 95% CI 0.75–1.05, based on 1115 events in 8019 patients.
Death	RR 0.91, 95% CI 0.74–1.12
Nonfatal myocardial infarction	RR 0.98, 95% CI 0.83–1.16
Nonfatal stroke	RR 0.78, 95% CI 0.63–0.98
Heart failure	RR 0.54, 95% CI 0.37–0.81
**2. Beta blocker compared to other antihypertensive agents** ^**1**^ (7 trials and *n* = 87,180 patients, range mean age 60.4 to 76 years)	
**Primary outcome:** composite cardiovascular outcome of death, nonfatal myocardial infarction or nonfatal stroke	Beta blockers were associated with a higher risk of events than were other antihypertensive agents (7405 events, RR 1.06, 95% CI 1.01–1.10).
Death	RR 1.05, 95% CI 0.99–1.11
Nonfatal myocardial infarction	RR 1.06, 95% CI 0.94–1.20
Nonfatal stroke	RR 1.18, 95% CI 1.07–1.30
Heart failure	RR 0.98, 95% CI 0.87–1.11

*Legend: RR* relative risk, *CI* confidence interval, ^1^p–values not reported

**Table 6:** Summary of study findings

Authors year	Findings			
Carlsson 2014 [43]	Prescription of selective or non-selective beta blockers did not affect mortality other than no treatment. Prescription of non-selective beta blockers was associated with lower mortality in sex-adjusted models Full regression model of the whole study sample adjusted for sex and all other covariates: non-selective beta blockers vs. beta 1-selective beta blockers HR 0.62 (95% CI: 0.41–0.95).			
	*men aged ≥80 y* *(HR (95* *%CI))*: beta 1-selective vs. no treatment: Model A: 1.01 (0.60–1.70) Model B: 1.09 (0.64–1.85) non-selective vs. no treatment: Model A: 0.47 (0.14–1.64) Model B: 0.53 (0.15–1.88) non-selective vs. beta 1-selective Model A: 0.42 (0.13–1.37) Model B: 0.39 (0.12–1.25)		*men all ages (HR (95%CI)):* beta 1-selective vs. no treatment: Model A: 0.99 (0.69–1.40) Model B: 1.12 (0.77–1.59) non-selective vs. no treatment: Model A: 0.55 (0.28–1.08) Model B: 0.68 (0.34–1.36) non-selective vs. beta 1-selective Model A. 0.57 (0.32–1.05) Model B: 0.54 (0.29–1.01)	
	*women aged ≥80 y(HR (95%CI)):* beta 1-selective vs. no treatment: Model A: 0.87 (0.58–1.31) Model B: 0.90 (0.59–1.38) non-selective vs. no treatment: Model A: 0.77 (0.36–1.64) Model B: 0.76 (0.35–1.64) non-selective vs. beta 1-selective Model A:0.88 (0.44–1.76) Model B: 0.86 (0.41–1.75)		*women all ages(HR (95%CI)):* beta 1-selective vs. no treatment Model A: 0.85 (0.60–1.20) Model B: 0.88 (0.62–1.25) non-selective vs. no treatment Model A: 0.62 (0.33–1.17) Model B. 0.66 (0.34–1.25) non-selective vs. beta 1-selective Model A: 0.76 (0.43–1.35) Model B: 0.73 (0.41–1.31)	
	Model A (prospensity score age group, cardiovascular comorbidity (diabetes, CHD, CHF and CVDs), educational level and marital status) Model B (propensity score including all variables in Model A and all the antihypertensive drugs, antithrombotics and statins (except the studied drug class).			
Collier 2011 [44]	Compared with the atenolol-based regimen, the amlodipine-based regimen reduced: - the relative risk of cardiovascular events more effectively in both age groups: by 17% in patients ≥65 years (hazard ratio: 0.83; 95% CI 0.75, 0.91; *P* < 0.01) and 15% in patients <65 years (hazard ratio 0.85; 95% CI 0.78, 0.95; *P* < 0.01) - cardiovascular mortality by 23% in the older group (hazard ratio 0.77; 95% CI 0.63, 0.94; *P* < 0.01) and by 24% in the younger group (hazard ratio 0.76; 95% CI 0.58, 1.00; *P* = 0.05) - fatal and nonfatal stroke by 30% in the older group (hazard ratio 0.70; 95% CI 0.59, 0.84; *P* < 0.01) and by nonsignificant 9% in the younger group (hazard ratio 0.91; 95% CI 0.71, 1.15; *P* = 0.42) - - significant in total of coronary endpoints, nonfatal MI (excluding silent MI) and fatal CHD in the younger, but not in the older group			
Coope 1986 [37]	Overall no significant difference in the total mortality was found neither in treatment nor in the control group. The rate of all deaths in the intervention group was 0.97 of that in the control group (95% CI 0.70–1.42).The rate of fatal stroke in the intervention group was 0.30 of that in the control group (95% CI 0.11–0.84) *p* < 0.025. Rate of all stroke in the intervention group was 0.58 of that in the control group (95% CI 0.35–0.96) *p* < 0.03. The subgroup analysis of patients by age (70–79 years) showed a similar reduction in total stroke in both groups, but the study was not large enough for these differences to be significant.			
Gelber 2013 [45]	Beta blocker use as the sole antihypertensive medication was associated with a lower risk of developing cognitive impairment (defined as a CASI [Cognitive Abilities Screening Instrument]) score < 74) compared with untreated men (IRR 0,69; 95% CI 0,50–0,94). Non- beta blocker drug combinations were also associated with a reduced risk (IRR 0,78; 95% CI 0,62–0,98). Cognitive decline (defined as a ≥ 9 point decrease in CASI score) occurred in 1167 men (53.1%). Beta blocker use was also associated with a trend toward a decreased risk of cognitive decline: model 2 IRR 0.78 (95% CI 0.61–1.00) for beta blocker use alone; 0.81 (95% CI 0.64–1.03) for beta blocker in combination with other drugs. None of the other drug categories was significantly associated with cognitive decline.			
Pepine 2003 [34]	No significant differences in primary outcome (first occurrence of all-cause death, nonfatal MI or nonfatal stroke) were seen between the calcium antagonist group and the non-calcium antagonist group (RR 0.98 CI 0.90–1.06). No significant differences were seen in these outcomes analysed individually. In the subgroup analysis of patients >70 years the primary outcome occurred in 596 of the 3694 patients of the calcium antagonist group (16.3%) and in 664 of the 3829 patients of the non- calcium antagonist group (17.34%). RR 0.93 (95% CI 0.84–1.03) [*p*-values missing]			
Ruwald 2012 [40]	In this post-hoc analysis of the LIFE study, patients were divided in subgroups of aged 66 or younger and aged 67 or older. In the older subgroup, losartan significantly reduced the risk of the composite primary endpoint of cardiovascular death, nonfatal stroke or nonfatal MI compared to atenolol (HR 0.79, 95% CI 0.69–0.91). In the younger subgroup the effect was not significant (HR 1.03, 95% CI 0.82–1.28). Further subdividing suggested a “cut-off age” of 71 years, above which losartan based treatment is better than atenolol based treatment.			
Testa 2014 [46]	Older adults taking atenolol showed a greater mortality and higher pulse arterial pressure values than those not taking atenolol (73.9% vs 55.0%; *p* = 0.047 and 74.7 ± 14.1 vs 63.0 ± 14.2 mmHg, *P* < 0.001, respectively). Cox regression analysis showed that atenolol use (HR 1.91; 95% CI 1.04–4.31; *p* = 0.04) and pulse arterial pressure (HR 1.02; 95% CI 1.01–1.03; *p* = 0.032) were predictive of long-term mortality.			
Matsuzaki 2011 Main trial [47]	For the subgroup ≥70 years: There are no statistical significant differences for the primary cardiovascular endpoint in people over 70 years regarding the three intervention arms.			
	**Primary cardiovascular composite endpoint:**			
	*Hazard ratio (95%CI)*		*P-value*	
	beta blocker/ARB: <70 years: 1.24 (0.72–2.12) ≥70 years: 1.21 (0.63–2.33) Overall population: 1.22 (0.80–1.85)		beta blocker/ARB: subgroup: 0.9671 overall: 0.3372	
	ARB/TD: <70 years: 1.59 (0.83–3.02) ≥70 years: 0.97 (0.50–1.91) Overall population: 1.26 (0.80–2.01)		ARB/TD: subgroup: 0.3017 overall: 0.3505	
	beta blocker/TD: <70 years: 1.96 (1.05–3.66) ≥70 years:1.18 (0.61–2.27) Overall population: 1.54 (0.98–2.41)		beta blocker/TD: subgroup: 0.2698 overall: 0.0567	
	The number of patients who discontinued the trial because of serious adverse events was 12 (1.1%), 11 (1.0%), and 11 (1.0%) in the benidipine-ARB, benidipine-beta blocker, and benidipine-thiazide groups, respectively. The percentage of adverse events was similar among the treatment groups: 505 (45.5%), 495 (45.5%), and 522 (47.7%) patients reported adverse events. The following adverse events occurred more frequently in another group or in two groups: bradycardia (benidipine-beta blocker,*P* < 0.0001), hyperkalemia (benidipine-ARB, *P* = 0.0395), vertigo (benidipine-b-blocker and benidipine-thiazide, *P* = 0.0188).			
Ogihara 2012 [41]	In this analysis of the COPE trial with 3293 patients in the subgroup of patients aged 65 years or older, benididpe + beta blocker reduced less fatal and non-fatal strokes than benidipine + TD (HR 2.74 (1.08–6-96) and benidipine +beta blocker was associated with more new onset diabetes than benidipine +ARB (HR 2.47 (1.03–5.91). There was no significant difference regarding the composite primary endpoint, cardiovascular endpoints, and all-cause mortality (benidipine plus beta blocker vs. benidipine plus angiotensine receptor blocker HR 0.99 (0.54–1.82); benidipine plus beta blocker vs. benidipine plus thiazide diuretic HR 1.34 (0.69–2.60).			
MRC 1992 [35] Main trial	Overall, the beta blocker group had significantly more withdrawals than the diuretic group for both suspected major side effects (beta blocker 30.2% (*n* = 333); diuretic 14.8% (*n* = 160); placebo 3.7% (*n* = 82), and inadequate blood pressure control (beta blocker *n* = 12; diuretic *n* = 1; placebo *n* = 175). After adjusting for baseline characteristics the diuretic group had significantly reduced risk of stroke (31% 95 CI 3% to 51%, *p* = 0.04), coronary events (44% 95 CI 21% to 60%, *p* = 0.0009), and all cardiovascular events (35% 95 CI 17% to 49%, *p* = 0.0005) compared with the placebo groups. The beta blocker group showed no significant reductions in these end points.			
	**Outcome, no. events**	**Diuretic** (*n* = 1081, 6290 patient years)	**Placebo** (*n* = 2213, 12,735 patient years)	**Relative risk per event/treatment (95% CI,** ***p*** **-value)** Based on own calculations:
	Stroke fatal	16	42	0.78 (0.44–1.38, *p* = 0.39)
	Stroke-nonfatal	29	92	0.65 (0.43–0.97, *p* = 0.04)
	Stroke total	45	134	0.69 (0.49–0.96, *p* = 0.03)
	Coronary events fatal	33	110	0.61 (0.42–0.90, *p* = 0.01)
	Coronary events-non-fatal	15	49	0.63 (0.35–1.11, *p* = 0.11)
	All cardiovascular events	107	309	0.71 (0.58–0.87, *p* = 0.0012)
	All cardiovascular deaths	66	180	0.75 (0.57–0.99, *p* = 0.04)
	All deaths	134	315	0.87 (0.72–1.05, *p* = 0.15)
		**Beta blocker (** *n* = 1102, 6330 patient years)	**Placebo** (*n* = 2213, 12,735 patient years)	
	Stroke fatal	21	42	1.00 (0.60–1.69, *p* = 0.99)
	Stroke-nonfatal	35	92	0.76 (0.52–1.11, *p* = 0.16)
	Stroke total	56	134	0.84 (0.62–1.14, *p* = 0.26)
	Coronary events fatal	52	110	0.95 (0.69–1.31, *p* = 0.75)
	Coronary events-non-fatal	28	49	1.15 (0.73–1.82, *p* = 0.56)
	All cardiovascular events	151	309	0.98 (0.82–1.18, *p* = 0.84)
	All cardiovascular deaths	95	180	1.06 (0.84–1.34, *p* = 0.63)
	All deaths	167	315	1.06 (0.90–1.27, *p* = 0.48)
Bird 1990 [36] Study based on the MRC trial population	**Blood pressure** (mean mmHg)	**Diuretic group**	**Beta blocker group**	**Placebo groups**
	1 month	152/80 (*n* = 635)	159/79 (*n* = 624)	166/85 (*n* = 1303)
	9 months	149/79 (*n* = 582)	156/79 (*n* = 481)	167/86 (1156)
	**Depression Questionnaire** (% of participants with depressed mood)			
	1 month	9.1 (*n* = 551)	11.0 (*n* = 554)	8.8 (*n* = 1147)
	9 months	10.4 (*n* = 550)	10.4 (*n* = 453)	9.2 (*n* = 1092)
	**Paired Associate Learning Test** (% of participants with a score ≤ 15)			
	1 month	21.0 (*n* = 593)	20.0 (*n* = 579)	20.0 (*n* = 1212)
	9 months	21.2 (*n* = 556)	19.9 (*n* = 485)	18.5 (*n* = 1116)
	**The Trial-Making Test** (seconds)			
	1 month, mean (SD)	54.3 (23.9) (*n* = 593)	54.7 (23.9) *n* = 580)	55.5 (27.7) (*n* = 1221)
	9 months, mean (SD)	52.4 (33.5) (*n* = 560)	53.1 (28.2) *n* = 460)	52.2 (25.6) (*n* = 1122)
	Antihypertensive treatment with either 25 mg hydrochlorothiazide and 2.5 mg amiloride or atenolol 50 mg for 9 months in a population aged between 65 and 74 years old with moderate raised blood pressure did not impair cognitive function, depression, or behavioural changes which would cause concern in confidants. The study did not find a linear relationship between blood pressure levels in their moderately elevated range and psychometric test scores, nor showed that lowering of blood pressure levels is associated with any impairment of performance on these tests			
Carr 2012 [42]	This secondary analysis of the MRC trial found evidence that atenolol does not perform as well as the hydrochlorothiazide plus amiloride (Moduretic ©) in terms of stroke prevention: Moduretic 41.6 number of events /1000 patient years, beta blocker 50.8% and placebo 60.5% (statistical tests missing). For stroke, we found that after adjusting for current systolic blood pressure variability in systolic blood pressure over time, as measured by the standard residual or root successive variance, contained significant prognostic capability: the rate ratio associated with an increase of 1 standard deviation in the standard residual was 1.25 95% CI 0.86–1.81 and 1.16 95% CI 0.85–1.59 for the root successive variance.			
Lever 1992 [48]	In this article reporting on the MRC trial, the beta blocker group showed no significant differences to placebo regarding the outcomes stroke, coronary events, all cardiovascular events and all-cause mortality.			
Lever 1993 [39]	In this article reporting on the MRC trial, a RCT with 4396 patients (mean age 70.3 years) randomized to beta blocker, diuretic or placebo, the beta blocker group showed no significant differences to placebo regarding the outcomes stroke, coronary events, all cardiovascular events and all-cause mortality.			

*Legend: RR* relative risk, *CI* confidence interval, *SD* standard deviation, *B* beta blocker, *C* comparator, *NR* not reported, *IRR* incidence risk ratio, *HR* hazard ratio, *TD* thiazide diuretic, *ARB* angiotensin receptor blocker, *ACE* angiotensin converting enzyme (inhibitor), *CHD* chronic heart disease, *CHF* chronic heart failure, *CVD* cardiovascular disease, *MI* myocardial infarction, *RCT* randomized controlled trial, *FU* Follow Up

#### Composite outcome of death, nonfatal stroke and nonfatal myocardial infarction

The meta-analysis [33] found that no reduced event rates for the composite outcome were found in beta blocker treatment groups compared to placebo in trials enrolling older adults (relative risk RR 0.89, 95% confidence interval CI 0.75–1.05). But in the comparison with other antihypertensive agents, a higher risk of events was found in the beta blocker- group (RR 1.06, 95% CI 1.01–1.10). In the subgroup analysis of one of the included randomized controlled trials [34], no significant difference in the first occurrence of the composite outcome was found between the verapamil-treatment-group and the atenolol-treatment-group (RR 0.93, 95% CI 0.84–1.03). Ruwald et al. [40] analysed the combined endpoint of cardiovascular death, nonfatal stroke and nonfatal myocardial infarction and found that in patients ≥67 years losartan significantly reduced the risk of this combined endpoint compared to atenolol (hazard ratio HR 0.79, 95% CI 0.69–0.91).

#### Mortality

The analysis of the included group of studies of the meta-analysis [33] found no appreciable impact on rates of death, neither comparing beta blocker treatment- and placebo- groups (RR 0.91, 95% CI 0.74–1.12), nor comparing beta blocker- and other antihypertensive agent- groups (RR 1.05, 95% CI 0.99–1.11) The MRC trial [35] confirms this results (RR 1.06, 95% CI 0.90–1.27, *p* = 0.48), as well as Ogihara et al.: all-cause mortality: benidipine plus beta blocker vs. benidipine plus angiotensine receptor blocker HR 0.99 (0.54–1.82); benidipine plus beta blocker vs. benidipine plus thiazide diuretic HR 1.34 (0.69–2.60) [41].Testa et al. [46] found that older adults taking atenolol showed higher mortality rates than those not taking atenolol (73.9% vs 55.0%; *p* = 0.047). Cox regression analysis of this paper showed that atenolol use was predictive for long term mortality (HR 1.91; 95% CI 1.04–4.31; *p* = 0.04). Carlsson et al. [43] found that the prescription of beta 1 selective or non-selective beta blockers did not affect mortality other than no treatment (for numbers, see Table 6). Further, the prescription of non-selective beta blockers compared to beta 1 selective beta blockers was associated with lower mortality in sex-adjusted models HR 0.62 (95% CI 0.41–0.95).

#### Cardiovascular events – including stroke

The analysis of the included group of studies of the meta-analysis [33] states that in older adults, beta blockers were associated with statistically significant reductions in nonfatal stroke (RR 0.78, 95% CI 0.63–0.98) and heart failure (RR 0.54, 95% CI 0.37–0.81) compared with placebo, but had no appreciable impact on rates of myocardial infarction (RR 0.98, 95% CI 0.83–1.16). Compared to other antihypertensive agents, beta blockers were associated with significantly higher rates of nonfatal stroke (RR 1.18, 95% CI 1.07–1.30), but not of heart failure (RR 0.98, 95% CI 0.87–1.11) and myocardial infarction (RR 1.06, 95% CI 0.94–1.20). The observational study of Collier et al. [44]compared an amlodipine- to a atenolol-based treatment and confirms these latter statements by showing that the amlodipine-based treatment reduced the relative risk of cardiovascular events more efficiently in older adults by 17% (HR 0.83; 95% CI 0.75–0.91; *p* < 0.01) and cardiovascular mortality by 23% (HR 0.77; 95% CI 0.63–0.94; *p* < 0.01). In this paper fatal and non-fatal stroke were also more efficiently reduced in the amlodipine-based treatment by 30% (HR 0.70; 95% CI 0.59–0.84; *p* < 0.01). The MRC trial [35] found no significant reduction in cardiovascular endpoints comparing beta blocker treatment with placebo in older adults: stroke total: RR 0.84 (95% CI 0.62–1.14, *p* = 0.26), all cardiovascular events: RR 0.98 (95% CI 0.82–1.18, *p* = 0.84). Carr et al. [42], a secondary analysis of the MRC trial found evidence that atenolol does not perform as well as the hydrochlorothiazide plus amiloride (Moduretic ©) in terms of stroke prevention: Moduretic 41.6 number of events /100 patient years, beta blocker 50.8% and placebo 60.5% (statistical tests missing). Matsuzaki et al. [47] analysed the composite endpoint of cardiovascular mortality and morbidity (see Table 3) and found, that there were no statistical significant differences regarding the analyses containing beta blockers: beta blocker compared to angiotensin receptor blocker HR 1.22 (95% CI 0.80–1.85, *p* = 0.3372) and beta blocker compared to thiazide diuretic HR 1.54 (95% CI 0.98–2.41, *p* = 0.0567).

Regarding the endpoint of total stroke, Coope et al. [37] analysed data comparing beta blocker treatment to no treatment in a subgroup analysis involving older adults >70 and found a similar reduction in both groups. They further state that the sample was not large enough for the differences to be significant (numbers were not available from the paper). Ogihara et al. [41] states that benidipine plus beta blocker reduced fatal and non-fatal stroke less than benidipine plus thiazide diuretics (HR 2.74; 95% CI 1.08–6.96).

#### Cognitive impairment/status

Bird [36], a secondary analysis of the the MRC trial [35] found that beta blocker treatment of moderate raised blood pressure in older adults did not impair cognitive function or produce symptoms or behaviour changes, which would cause concern in confidants of treated patients. Gelber et al. [45] found that beta blocker use as the sole antihypertensive medication was associated with a lower risk of developing cognitive impairment defined as a CASI (Cognitive Abilities Screening Instrument) score < 74) compared with untreated men (incidence rate ratio IRR 0.69; 95% CI 0.50–0.94). Beta blocker use was also associated with a trend toward a decreased risk of cognitive decline (defined as a ≥ 9 point decrease in CASI score): adjusted analysis IRR 0.78 (95% CI 0.61–1.00) for beta blocker use alone; IRR 0.81 (95% CI 0.64–1.03) for beta blocker in combination with other drugs. None of the other drug categories was significantly associated with cognitive decline.

#### Adverse events

The Matsuzaki trial [47] reported about adverse events. The number of patients who discontinued the trial because of serious adverse events was 12 (1.1%), 11 (1.0%), and 11 (1.0%) in the benidipine-ARB, benidipine-b-blocker, and benidipine-thiazide groups, respectively. The percentage of adverse events was similar among the treatment groups: 505 (45.5%), 495 (45.5%), and 522 (47.7%) of the patients reported adverse events. Bradycardia (benidipine-b-blocker, *p* < 0.0001) and vertigo (benidipine-b-blocker and benidipine- thiazide, *p* = 0.0188) occurred more frequently in the beta blocker group.

### Risk of bias - meta-analysis

A summary of risk of bias in the meta-analysis [33] is presented in Table 2. The main limitations contributing to a risk of bias were related to methodology issues. The literature search was done without using appropriate search strings and only two data bases were used, namely MEDLINE and the Cochrane library. A quality appraisal of included studies was lacking. The review process was unclear and a list of excluded studies with reason for exclusion was missing. Risk of publication bias was not assessed.

### Risk of bias - Clinical trials

**Fig. 2:**
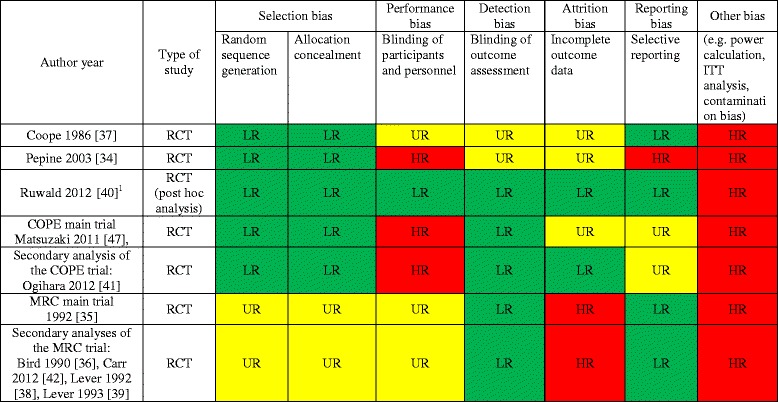
Quality appraisal for intervention studies. RCT = randomized controlled trial, LR = low risk of bias, HR = high risk of bias, UR = unclear (insufficient information to permit judgement of low risk or high risk), ITT = intention-to-treat analysis, COPE = Combination Therapy of Hypertension to Prevent Cardiovascular Events, MRC = Medical Research Council. ^1^ Risk of bias assessed on study protocol [56]

A summary of risk of bias for intervention studies is presented in Fig. 2. The main limitations contributing to risk of bias were related to the design (e.g. inadequate randomisation, blinding, attrition bias and selective reporting). Other potential sources of bias were related to intention-to-treat analysis, contamination bias, sample size and power calculation. In two studies a high crossover rate between groups was registered [34, 37]. Detailed information of the risk of bias is available in Additional file 4.

### Risk of bias - Observational studies

Quality appraisal of the four observational studies is shown in Table 7. Three studies can be judged as being of fairly good quality. One study reported on most of the CASP items insufficiently and cannot be considered of high quality.

**Table 7:** Quality appraisal for observational studies

Author year	1. Focused issue	2. Appropriate method	3. Recruitment	4. Selection of controls	5. Exposure measured	6. Outcome measured	7. Identified confounding factors?	8. Confounding factors in design/analysis	9. Follow up complete	10. Follow up long	11. Believe results	12. Results be applied	13. Results fit evidence
Carlsson 2014 [43]	u	y	y	y	y	y	u	y	y	y	y	y	y
Collier 2011 [44]	y	y	y	y	y	y	u	y	u	n	n	y	u
Gelber 2013 [45]	y	n	u	u	y	y	u	y	n	y	n	n	na
Testa 2014 [46]	y	y	y	y	y	y	u	y	y	y	u	y	u

*y* yes, *n* no, *u* unclear, *na* not applicable

### Additional references of interest for the development of recommendations

We found nine additional references that were taken into consideration for the development of the recommendations: three Cochrane reviews [17, 49, 50], one update of a Cochrane review [22], one meta-analysis [33], two evidence based recommendation paper [51, 52], one evidence based guideline [38] and one randomized controlled trial [53]. Details of the studies can be seen in Additional file 2. These references did not meet the inclusion criteria of our systematic review because of the age of the included participants. They were counted as being relevant to recommendations principally because they provided information about the risks and benefits of beta blockers for the management of hypertension in younger populations.

Three of the additional references evaluated atenolol [50, 53, 54]. Atenolol was reported to be associated with an increased risk of stroke compared with other antihypertensive agents [54] and to be less effective at preventing cardiovascular morbidity and death than losartan [53]. However, in a systematic review no statistical differences were found between atenolol and placebo regarding the risk of fatal and non-fatal stroke, major vascular events, death from all causes, and death from cardiovascular causes [50]. The other five additional references evaluated beta blockers [17, 38, 49, 51, 52] in general. Although the risk for stroke was lower for beta blockers compared to placebo, there was an increase in stroke in beta blockers compared to calcium channel blockers and renin-angiotensin system inhibitors [17]. This finding is in the same direction as the results reported by Khan et al. [33] for the group of studies enrolling older patients. Total mortality and cardiovascular disease were also higher for beta blockers compared to calcium channel blockers [17]. Furthermore, it was reported that second line beta blockers reduced blood pressure but with little or no effect on pulse pressure compared to thiazides [46]. The Canadian Hypertension Education Program papers were the only sources that recommended beta blockers as first line therapy for patients <60 year with coronary artery disease [37].

These additional references provide preliminary evidence that certain risks like fatal and non-fatal stroke, major vascular events, cardiovascular morbidity, death from all causes, and death from cardiovascular causes may be increased with the use of beta blockers compared to other antihypertensive treatments. The uncertainties regarding these risks imply that independently from the age group beta blockers should be prescribed with caution for the treatment of hypertension.

### Recommendations

We developed recommendations following a standardised schema and reflecting the strength and the quality of the evidence. From the results of our systematic review and the additional “references of interest” we were able to develop three recommendations in two meetings by AV (researcher and clinician), YVM (researcher) and ARG (researcher and geriatrician).AS participated in one of these as a senior clinician and researcher. The three recommendations were later discussed and confirmed with IK and MMV for their inclusion in the Comprehensive Medication Review (CMR) tool, which was developed within the PRIMA-eDS project.

The three recommendations we developed are about the discontinuation of beta blocker use in older adults with hypertension (Table 1). The recommendations were considered as strong recommendations. The quality was downgraded to low for indirectness.

## Discussion

This systematic review aimed to explore the effectiveness and safety of beta blockers for the management of hypertension in older adults (≥ 65 years). A further aim was to develop recommendations on when the use of beta blockers could be discontinued or its dose could be reduced in the treatment of hypertension in older adults.

### Summary of main results

One meta-analysis, four randomized controlled trials, six secondary analyses (post hoc analyses) of randomized controlled trials, and four observational studies were included in our systematic review. The included studies were heterogeneous in terms of study designs, interventions, settings, participants, reporting and definitions of outcome measurements, as well as length of follow-up. The included studies reported on mortality, cardiovascular events including stroke, cognitive impairment or cognitive status and adverse events. We did not find studies analysing the effect on quality of life, life expectancy, hospitalisation, functional impairment or functional status, renal failure, or safety endpoints. The results of the included studies suggest that beta blockers appear to increase the risk of death, stroke and myocardial infarction analysed together as a composite outcome if compared to other antihypertensive agents [31]. Further, beta blockers showed no benefit compared to other antihypertensive agents or placebo regarding the single outcome of mortality. They appear to be less effective than other antihypertensive agents in reducing cardiovascular events. Regarding the endpoint of cognitive impairment/decline, there seems to be a trend towards a decreased risk associated with beta blocker use versus no treatment. Regarding the single endpoint of stroke we found contradictory results.

In addition to the results of the effectiveness of beta blockers identified in the present review, also some issues regarding medication safety have to be risen. First, high cross-over-rates occurred in two studies [32, 34], where a high cross over from the beta blocker group to the comparison group occurred. Pepine et al. [32] gave the following possible reasons for this: physician bias against beta blockers, and suspected higher metabolic complications or previous intolerances against beta blockers.

Second, in another study [33], withdrawal of patients due to suspected major side effects and inadequate blood pressure control in the beta blocker group was significantly higher than in the comparison group.

Regarding the quality of the included studies and the interpretation of the results, a major limitation was that the results are partly based on subgroup analyses (3 out of 5 studies) [31, 32, 34] and secondary analyses of randomized controlled trials [36, 39–42, 48]. Subgroup analyses and secondary analyses in general are used for generating hypotheses and not for testing hypotheses. The included meta-analysis has some major limitations and it can be argued that the systematic review on which it is based does not fulfil the criteria for a systematic review [19]. Only three of the 11 studies included in the subgroup analysis of older people in the meta-analysis were considered eligible for our review. The reporting of the participants’ characteristics was very poor with regard to their comorbidities, use of concomitant drugs, frailty, and cognitive status. Thus, the generalizability of the results to the heterogeneous group of older people is limited as the evidence is indirect. However, it could be expected that concerns with regard to safety and benefits of beta blockers for the management of hypertension identified in the present study may be even higher for frailer older people.

### Strengths and limitations

We conducted this systematic review following an adaption of the standard methodology recommended by the Cochrane collaboration [19] and the PRISMA statement [20]. The search strategy comprised a stepwise approach searching first for systematic reviews and meta-analysis (searches 1 and 2), and then for additional relevant controlled intervention and observational studies (searches 3A and 3B). We restricted search 3B to the period 2011–2016, as earlier literature had already been covered in the process of searches 1, 2 and 3A. Taking into account that the new update of the Cochrane Review of Wiysonge published in 2017 [22] did not identify any additional studies that would be eligible for our review (and the review does not apply any age restrictions), it is very unlikely that any eligible studies have been missed by our search strategy. We excluded a large number of studies because they were either not focussing on beta blockers or they had been carried out in younger populations (< 65 years). Our search strategy included specific terms for older adults. This might have limited our ability to capture all studies that include evidence on older adults carrying a risk of missing studies like in a rapid review. But we believe our stepwise approach overcomes some of the limitations of a rapid review, for which there is a lack of published guidelines or explicit methods [51, 52]. Unlike our review, some rapid reviews do not have clear research questions including participants, interventions, comparisons, outcomes, and study design (PICOS) [51].

Three of the four individual randomized controlled trials we included [34, 35, 37] also formed part of the included subgroup analysis of the meta-analysis [33]. Although this does carry a risk of “double-counting”, we decided to include both in our systematic review as the overlap was small (3 out of 11 trials in the meta-analysis) and the meta-analysis did not examine these studies to the same level of detail as we are doing in our systematic review.

Two of the secondary analyses [39, 48] did not really provide any new data and they also could have been treated as duplicates but as they formally met our inclusion criteria we included them as secondary analyses of the MRC study.

In our systematic review we only found studies specifically addressing Atenolol. It therefore remains unclear whether the results of these studies are also applicable for newer beta blockers such as metoprolol or bisoprolol.

### Implications for future research

Older adults comprise the great majority of consumers of drugs used in the treatment of chronic diseases such as hypertension. Yet, there has been little work to systematically assess the research evidence for usage in this population (≥65 years with polypharmacy and/or multimorbidity). Research groups should focus on clinically relevant outcomes i.e. mortality, cardiovascular diseases (including stroke) or hospital admissions (drug related) whenever applicable, as these are critical in the context of beta blocker treatment in hypertension. We also want to mention that there are a number of other important endpoints, e.g. quality of life, functional and cognitive status which should be considered especially in studies on older populations. A proper power calculation and definition of a primary endpoint (single or composite) is important to show an effect and to avoid multiple testing. In most studies important information is lacking in the methods section. Study authors should provide a clear and comprehensive description of the population (including comorbidities), study design and intervention. We highly recommend researchers to use the Consolidated Standards of Reporting Trials statement (CONSORT) [55] to improve the quality of reporting of randomized controlled trials.

## Conclusions

This is one of the first systematic reviews exploring the risks and benefits of beta blockers in treating hypertension in aged populations (>65 years) on patient relevant outcomes. This study highlights the limited number of studies that have been carried out on this issue. Furthermore, the quality of current evidence to interpret the benefits of beta blockers in hypertension is generally weak. It cannot be recommended to use beta blockers in older adults as a first line agent for hypertension. Further studies of good quality should analyse the risks and benefits of beta blockers in older people with hypertension taking into account their comorbidities, use of concomitant medications, functional and cognitive status, and evaluating clinically relevant outcomes for this population.

## Additional files

Additional file 1: Search strings used in the literature database search. (DOC 1066 kb)

Additional file 2: Additional evidence for recommendations. (DOCX 19 kb)

Additional file 3: List of excluded studies. (XLSX 46 kb)

Additional file 4: Risk of bias – Clinical trials. (DOCX 71 kb)

## Abbreviations

ALT: Anne-Lisa Teichmann
AMSTAR: A Measurement Tool to Assess Systematic Reviews
AR: Anja Rieckert
ARG: Anna Renom-Guiteras
AS: Andreas Sönnichsen
AV: Anna Vögele
AW: Adrine Woodham
BF: Barbara Faller
CASP: Critical Appraisal Skills Programme
CMR: Comprehensive Medication Review
DARE: Database of Abstracts or Reviews of Effects
DR: David Reeves
EMBASE: Excerpta Medica dataBASE
GRADE: Grading of Recommendations Assessment, Development and Evaluation
HTA: Health Technology Assessment
IK: Ilkka Kunnamo
IPA: International Pharmaceutical Abstracts
LS: Lisa Schlender
MMV: Minna Marttila Vaara
PICOS: Population, Intervention, Comparison, Outcomes and Study design
PRIMA-eDS: Polypharmacy in chronic diseases: Reduction of Inappropriate Medication and Adverse drug events in elderly populations by electronic Decision Support
PRISMA: Preferred Reporting Items for Systematic Reviews and Meta-Analyses
SW: Sabine Weißbach
TJ: Tim Johansson
YVM: Yolanda Veronica Martinez
