# Supplementary material for: Effectiveness and safety of beta blockers in the management of hypertension in older adults: a systematic review to help reduce inappropriate prescribing
Source: BMC Geriatr. 2017 Oct 16;17(Suppl 1):224. doi: 10.1186/s12877-017-0575-4 (PMC5647554; doi:10.1186/s12877-017-0575-4)
Supplement: Supplementary file 1 — Search strings used in the literature database search. (DOC 1066 kb) [file 12877_2017_575_MOESM1_ESM.doc]

**Additional file 1:** Search strings used in the literature database searches.

**EBM Reviews - Database of Abstracts of Reviews of Effects 3rd Quarter 2013**

**(11.09.2013)**

**1st search: Beta-Blocking Agents in the management of Hypertension**

**Population AND Condition AND Intervention AND Outcome AND Limits, Study designs**

| [**# ▲**](http://ovidsp.uk.ovid.com/sp-3.8.1a/ovidweb.cgi?&S=DCDLPDGALHHFOEDMFNOKEBEGNLKJAA00&Sort+Sets=descending) | | **Searches** | **Results** | |
| --- | --- | --- | --- | --- |
| **Population** | | | | |
|  | geriatrics.mp. or exp geriatrics/ | | | 36 |
|  | geriatric patient.mp. | | | 1 |
|  | geriatric*.mp. | | | 258 |
|  | (elder$ or geriatric$).ab,ti. | | | 197 |
|  | elder*.mp. | | | 625 |
|  | frail elderly.mp. or exp frail elderly/ | | | 55 |
|  | aged.mp. or exp Aged/ | | | 4585 |
|  | old*.mp. | | | 1679 |
|  | old* adult*.mp. | | | 243 |
|  | old* people*.mp. | | | 156 |
|  | >65.mp. | | | 792 |
|  | over 65.mp. | | | 60 |
|  | or/1-12 | | | 5844 |
| **Condition** | | | | |
|  | high blood pressure.mp. | | | 26 |
|  | hypertension.mp. or exp hypertension/ | | | 950 |
|  | or/14-15 | | | 961 |
| **Intervention** | | | | |
|  | beta-blocker.mp. | | | 87 |
|  | beta blocking agent*.mp. | | | 5 |
|  | Adrenergic beta-Antagonists.mp. or exp Adrenergic beta-Antagonists/ | | | 141 |
|  | alprenolol.mp. | | | 2 |
|  | oxprenolol.mp. | | | 15 |
|  | pindolol.mp. | | | 27 |
|  | propranolol.mp. | | | 68 |
|  | timolol.mp. | | | 31 |
|  | sotalol.mp. | | | 32 |
|  | nadolol.mp. | | | 16 |
|  | mepindolol.mp. | | | 0 |
|  | carteolol.mp. | | | 1 |
|  | tertatolol.mp. | | | 0 |
|  | bopindolol.mp. | | | 2 |
|  | bupranolol.mp. | | | 1 |
|  | penbutolol.mp. | | | 2 |
|  | cloranolol.mp. | | | 0 |
|  | practolol.mp. | | | 5 |
|  | metoprolol.mp. | | | 72 |
|  | atenolol.mp. | | | 76 |
|  | acebutolol.mp. | | | 14 |
|  | betaxolol.mp. | | | 9 |
|  | bevantolol.mp. | | | 0 |
|  | bisoprolol.mp. | | | 37 |
|  | celiprolol.mp. | | | 5 |
|  | esmolol.mp. | | | 10 |
|  | epanolol.mp. | | | 3 |
|  | s-atenolol.mp. | | | 0 |
|  | nebivolol.mp. | | | 15 |
|  | talinolol.mp. | | | 0 |
|  | labetalol | | | 17 |
|  | carvedilol | | | 37 |
|  | or/17-48 | | | 305 |
| **Outcome** | | | | |
|  | mortality.mp. or exp mortality/ | | | 3813 |
|  | quality of life.mp. or exp quality of life/ | | | 1901 |
|  | QOL.mp. | | | 67 |
|  | cardiovascular event.mp. | | | 49 |
|  | myocardial infarction.mp. | | | 1093 |
|  | stroke.mp. | | | 1204 |
|  | hospitalization.mp. or exp hospitalization/ | | | 237 |
|  | hospitalisation.mp. or exp hospitalisation/ | | | 488 |
|  | life expectancy.mp. | | | 38 |
|  | cognitive impairment.mp. | | | 118 |
|  | cognitive status.mp. | | | 13 |
|  | functional status.mp. | | | 191 |
|  | functional impairment.mp. | | | 29 |
|  | renal failure.mp. | | | 168 |
|  | renal insufficiency.mp. or exp renal insufficiency/ | | | 97 |
|  | adverse drug event.mp. | | | 1 |
|  | adverse effects.mp. or exp adverse effects/ | | | 5162 |
|  | drug toxicity.mp. or exp drug toxicity/ | | | 67 |
|  | safety.mp. | | | 2721 |
|  | patient safety.mp. or exp patient safety/ | | | 68 |
|  | falls.mp. | | | 168 |
|  | delirium.mp. or exp delirium/ | | | 67 |
|  | or/50-71 | | | 10827 |
| **Limits, Study designs** | | | | |
|  | (systematic review.ti. or meta-analysis.pt. or meta-analysis.ti. or systematic literature review.ti. or (systematic review.ti,ab. and review.pt.) or consensus development conference.pt. or practice guideline.pt. or cochrane database of systematic reviews.jn. or acp journal club.jn. or health technology assessment winchester england.jn. or evidence report technology assessment summary.jn. or drug class reviews.ti. or (clinical guideline and management).tw. or ((evidence based.ti. or evidence-based medicine.sh. or best practice*.ti. or evidence synthesis.ti,ab.) and (((review.pt. or diseases category.mp. or behaviour.sh.) and behavior mechanisms.mp.) or therapeutics.sh. or evaluation studies.pt. or validation studies.pt. or guideline.pt. or pmcbook.mp.)) or (((systematic or systematically).tw. or critical.ti,ab. or study selection.tw. or ((predetermined or inclusion) and criteri*).tw. or exclusion criteri*.tw. or main outcome measures.tw. or standard of care.tw. or standards of care.tw.) and ((survey or surveys).ti,ab. or overview*.tw. or review.ti,ab. or reviews.ti,ab. or search*.tw. or handsearch.tw. or analysis.ti,ab. or critique.ti,ab. or appraisal.tw. or (reduction.tw. and (risk.sh. or risk.tw.) and (death or recurrence).mp.)) and ((literature or articles or publications or publication or bibliography or bibliographies or published).ti,ab. or unpublished.tw. or citation.tw. or citations.tw. or database.ti,ab. or internet.ti,ab. or textbooks.ti,ab. or references.tw. or scales.tw. or papers.tw. or datasets.tw. or trials.ti,ab. or meta-analy*.tw. or (clinical and studies).ti,ab. or treatment outcome.sh. or treatment outcome.tw. or pmcbook.mp.))) not (letter or newspaper article or comment).pt. | | | 22082 |
| **Population AND Condition AND Intervention AND Outcome AND Limits, Study designs** | | | | |
|  | 13 and 16 and 49 and 72 and 73 | | | **52** |

**No** **Limits**

**Total hits: 52**

**NO DUPLICATES**

**EBM Reviews - Cochrane Database of Systematic Reviews 2005 to July 2013**

**(11.09.2013)**

**1^st^ search: Beta-Blocking Agents in the management of Hypertension**

**Population AND Condition AND Intervention AND Outcome AND Limits, Study designs**

| [**# ▲**](http://ovidsp.uk.ovid.com/sp-3.8.1a/ovidweb.cgi?&S=DCDLPDGALHHFOEDMFNOKEBEGNLKJAA00&Sort+Sets=descending) | | **Searches** | **Results** | |
| --- | --- | --- | --- | --- |
| **Population** | | | | |
|  | geriatrics.mp. or exp geriatrics/ | | | 55 |
|  | geriatric patient.mp. | | | 1 |
|  | geriatric*.mp. | | | 212 |
|  | (elder$ or geriatric$).ab,ti. | | | 84 |
|  | elder*.mp. | | | 930 |
|  | frail elderly.mp. or exp frail elderly/ | | | 37 |
|  | aged.mp. or exp Aged/ | | | 2361 |
|  | old*.mp. | | | 3410 |
|  | old* adult*.mp. | | | 233 |
|  | old* people*.mp. | | | 353 |
|  | >65.mp. | | | 1954 |
|  | over 65.mp. | | | 1954 |
|  | or/1-12 | | | 4962 |
| **Condition** | | | | |
|  | high blood pressure.mp. | | | 225 |
|  | hypertension.mp. or exp hypertension/ | | | 1233 |
|  | or/14-15 | | | 1263 |
| **Intervention** | | | | |
|  | beta-blocker.mp. | | | 95 |
|  | beta blocking agent*.mp. | | | 11 |
|  | Adrenergic beta-Antagonists.mp. or exp Adrenergic beta-Antagonists/ | | | 42 |
|  | alprenolol.mp. | | | 13 |
|  | oxprenolol.mp. | | | 26 |
|  | pindolol.mp. | | | 31 |
|  | propranolol.mp. | | | 78 |
|  | timolol.mp. | | | 36 |
|  | sotalol.mp. | | | 28 |
|  | nadolol.mp. | | | 27 |
|  | mepindolol.mp. | | | 9 |
|  | carteolol.mp. | | | 15 |
|  | tertatolol.mp. | | | 7 |
|  | bopindolol.mp. | | | 8 |
|  | bupranolol.mp. | | | 11 |
|  | penbutolol.mp. | | | 11 |
|  | cloranolol.mp. | | | 5 |
|  | practolol.mp. | | | 14 |
|  | metoprolol.mp. | | | 50 |
|  | atenolol.mp. | | | 53 |
|  | acebutolol.mp. | | | 21 |
|  | betaxolol.mp. | | | 19 |
|  | bevantolol.mp. | | | 7 |
|  | bisoprolol.mp. | | | 25 |
|  | celiprolol.mp. | | | 17 |
|  | esmolol.mp. | | | 21 |
|  | epanolol.mp. | | | 8 |
|  | s-atenolol.mp. | | | 0 |
|  | nebivolol.mp. | | | 15 |
|  | talinolol.mp. | | | 7 |
|  | labetalol.mp. | | | 29 |
|  | carvedilol.mp. | | | 28 |
|  | or/17-48 | | | 187 |
| **Outcome** | | | | |
|  | mortality.mp. or exp mortality/ | | | 3805 |
|  | quality of life.mp. or exp quality of life/ | | | 3993 |
|  | QOL.mp. | | | 569 |
|  | cardiovascular event.mp. | | | 69 |
|  | myocardial infarction.mp. | | | 674 |
|  | stroke.mp. | | | 1053 |
|  | hospitalization.mp. or exp hospitalization/ | | | 472 |
|  | hospitalisation.mp. or exp hospitalisation/ | | | 1237 |
|  | life expectancy.mp. | | | 269 |
|  | cognitive impairment.mp. | | | 342 |
|  | cognitive status.mp. | | | 36 |
|  | functional status.mp. | | | 356 |
|  | functional impairment.mp. | | | 133 |
|  | renal failure.mp. | | | 468 |
|  | renal insufficiency.mp. or exp renal insufficiency/ | | | 119 |
|  | adverse drug event.mp. | | | 4 |
|  | adverse effects.mp. or exp adverse effects/ | | | 4676 |
|  | drug toxicity.mp. or exp drug toxicity/ | | | 68 |
|  | safety.mp. | | | 3424 |
|  | patient safety.mp. or exp patient safety/ | | | 93 |
|  | falls.mp. | | | 403 |
|  | delirium.mp. or exp delirium/ | | | 103 |
|  | or/50-71 | | | 7360 |
| **Limits, Study designs** | | | | |
|  | (systematic review.ti. or meta-analysis.pt. or meta-analysis.ti. or systematic literature review.ti. or (systematic review.ti,ab. and review.pt.) or consensus development conference.pt. or practice guideline.pt. or cochrane database of systematic reviews.jn. or acp journal club.jn. or health technology assessment winchester england.jn. or evidence report technology assessment summary.jn. or drug class reviews.ti. or (clinical guideline and management).tw. or ((evidence based.ti. or evidence-based medicine.sh. or best practice*.ti. or evidence synthesis.ti,ab.) and (((review.pt. or diseases category.mp. or behaviour.sh.) and behavior mechanisms.mp.) or therapeutics.sh. or evaluation studies.pt. or validation studies.pt. or guideline.pt. or pmcbook.mp.)) or (((systematic or systematically).tw. or critical.ti,ab. or study selection.tw. or ((predetermined or inclusion) and criteri*).tw. or exclusion criteri*.tw. or main outcome measures.tw. or standard of care.tw. or standards of care.tw.) and ((survey or surveys).ti,ab. or overview*.tw. or review.ti,ab. or reviews.ti,ab. or search*.tw. or handsearch.tw. or analysis.ti,ab. or critique.ti,ab. or appraisal.tw. or (reduction.tw. and (risk.sh. or risk.tw.) and (death or recurrence).mp.)) and ((literature or articles or publications or publication or bibliography or bibliographies or published).ti,ab. or unpublished.tw. or citation.tw. or citations.tw. or database.ti,ab. or internet.ti,ab. or textbooks.ti,ab. or references.tw. or scales.tw. or papers.tw. or datasets.tw. or trials.ti,ab. or meta-analy*.tw. or (clinical and studies).ti,ab. or treatment outcome.sh. or treatment outcome.tw. or pmcbook.mp.))) not (letter or newspaper article or comment).pt. | | | 8328 |
| **Population AND Condition AND Intervention AND Outcome AND Limits, Study designs** | | | | |
|  | 13 and 16 and 49 and 72 and 73 | | | **78** |

**No** **Limits**

**Total hits: 78**

**No duplicates**

**Embase 1974 to 2013 October 16 (17.10.2013)**

**2nd search: Beta-Blocking Agents in the management of Hypertension**

**Population AND Condition AND Intervention AND Outcome AND Limits, Study designs**

| [**# ▲**](http://ovidsp.uk.ovid.com/sp-3.8.1a/ovidweb.cgi?&S=DCDLPDGALHHFOEDMFNOKEBEGNLKJAA00&Sort+Sets=descending) | | **Searches** | **Results** | |
| --- | --- | --- | --- | --- |
| **Population** | | | | |
|  | geriatrics.mp. or exp geriatrics/ | | | 49328 |
|  | geriatric patient.mp. | | | 15233 |
|  | geriatric*.mp. | | | 109775 |
|  | (elder$ or geriatric$).ab,ti. | | | 264950 |
|  | elder*.mp. | | | 261420 |
|  | frail elderly.mp. or exp frail elderly/ | | | 7051 |
|  | aged.mp. or exp Aged/ | | | 3075406 |
|  | old*.mp. | | | 1236537 |
|  | old* adult*.mp. | | | 43626 |
|  | old* people*.mp. | | | 23162 |
|  | >65.mp. | | | 366563 |
|  | over 65.mp. | | | 7150 |
|  | or/1-12 | | | 4219888 |
| **Condition** | | | | |
|  | high blood pressure.mp. | | | 14291 |
|  | hypertension.mp. or exp hypertension/ | | | 624673 |
|  | or/14-15 | | | 626771 |
| **Intervention** | | | | |
|  | beta-blocker.mp. | | | 14090 |
|  | beta blocking agent*.mp. | | | 2604 |
|  | Adrenergic beta-Antagonists.mp. or exp Adrenergic beta-Antagonists/ | | | 238433 |
|  | alprenolol.mp. | | | 5096 |
|  | oxprenolol.mp. | | | 5507 |
|  | pindolol.mp. | | | 10800 |
|  | propranolol.mp. | | | 94064 |
|  | timolol.mp. | | | 12571 |
|  | sotalol.mp. | | | 11335 |
|  | nadolol.mp. | | | 4966 |
|  | mepindolol.mp. | | | 370 |
|  | carteolol.mp. | | | 1471 |
|  | tertatolol.mp. | | | 354 |
|  | bopindolol.mp. | | | 302 |
|  | bupranolol.mp. | | | 1007 |
|  | penbutolol.mp. | | | 911 |
|  | cloranolol.mp. | | | 62 |
|  | practolol.mp. | | | 5669 |
|  | metoprolol.mp. | | | 28462 |
|  | atenolol.mp. | | | 27539 |
|  | acebutolol.mp. | | | 4982 |
|  | betaxolol.mp. | | | 3054 |
|  | bevantolol.mp. | | | 232 |
|  | bisoprolol.mp. | | | 6423 |
|  | celiprolol.mp. | | | 1435 |
|  | esmolol.mp. | | | 3763 |
|  | epanolol.mp. | | | 109 |
|  | s-atenolol.mp. | | | 52 |
|  | nebivolol.mp. | | | 2419 |
|  | talinolol.mp. | | | 651 |
|  | labetalol.mp. | | | 8817 |
|  | carvedilol.mp. | | | 10370 |
|  | or/17-48 | | | 245556 |
| **Outcome** | | | | |
|  | mortality.mp. or exp mortality/ | | | 870068 |
|  | quality of life.mp. or exp quality of life/ | | | 296293 |
|  | QOL.mp. | | | 30472 |
|  | cardiovascular event.mp. | | | 3403 |
|  | myocardial infarction.mp. | | | 185149 |
|  | stroke.mp. | | | 249263 |
|  | hospitalization.mp. or exp hospitalization/ | | | 255712 |
|  | hospitalisation.mp. or exp hospitalisation/ | | | 14632 |
|  | life expectancy.mp. | | | 39320 |
|  | cognitive impairment.mp. | | | 39510 |
|  | cognitive status.mp. | | | 3749 |
|  | functional status.mp. | | | 32982 |
|  | functional impairment.mp. | | | 12687 |
|  | renal failure.mp. | | | 101191 |
|  | renal insufficiency.mp. or exp renal insufficiency/ | | | 232771 |
|  | adverse drug event.mp. | | | 693 |
|  | adverse effects.mp. or exp adverse effects/ | | | 108688 |
|  | drug toxicity.mp. or exp drug toxicity/ | | | 82468 |
|  | safety.mp. | | | 605710 |
|  | patient safety.mp. or exp patient safety/ | | | 56206 |
|  | falls.mp. | | | 36736 |
|  | delirium.mp. or exp delirium/ | | | 19532 |
|  | or/50-71 | | | 2615292 |
| **Limits, Study designs** | | | | |
|  | (systematic review.ti. or meta-analysis.pt. or meta-analysis.ti. or systematic literature review.ti. or (systematic review.ti,ab. and review.pt.) or consensus development conference.pt. or practice guideline.pt. or cochrane database of systematic reviews.jn. or acp journal club.jn. or health technology assessment winchester england.jn. or evidence report technology assessment summary.jn. or drug class reviews.ti. or (clinical guideline and management).tw. or ((evidence based.ti. or evidence-based medicine.sh. or best practice*.ti. or evidence synthesis.ti,ab.) and (((review.pt. or diseases category.mp. or behaviour.sh.) and behavior mechanisms.mp.) or therapeutics.sh. or evaluation studies.pt. or validation studies.pt. or guideline.pt. or pmcbook.mp.)) or (((systematic or systematically).tw. or critical.ti,ab. or study selection.tw. or ((predetermined or inclusion) and criteri*).tw. or exclusion criteri*.tw. or main outcome measures.tw. or standard of care.tw. or standards of care.tw.) and ((survey or surveys).ti,ab. or overview*.tw. or review.ti,ab. or reviews.ti,ab. or search*.tw. or handsearch.tw. or analysis.ti,ab. or critique.ti,ab. or appraisal.tw. or (reduction.tw. and (risk.sh. or risk.tw.) and (death or recurrence).mp.)) and ((literature or articles or publications or publication or bibliography or bibliographies or published).ti,ab. or unpublished.tw. or citation.tw. or citations.tw. or database.ti,ab. or internet.ti,ab. or textbooks.ti,ab. or references.tw. or scales.tw. or papers.tw. or datasets.tw. or trials.ti,ab. or meta-analy*.tw. or (clinical and studies).ti,ab. or treatment outcome.sh. or treatment outcome.tw. or pmcbook.mp.))) not (letter or newspaper article or comment).pt. | | | 185394 |
| **Population AND Condition AND Intervention AND Outcome AND Limits, Study designs** | | | | |
|  | 13 and 16 and 49 and 72 and 73 | | | **177** |

**No** **Limits**

**Total hits: =177**

**No. of duplicates = 26**

**Ovid MEDLINE(R) 1946 to October Week 1 2013, Ovid MEDLINE(R) In-Process & Other Non-Indexed Citations October 01, 2013 (17.10.2013)**

**2nd search: Beta-Blocking Agents in the management of Hypertension**

**Population AND Condition AND Intervention AND Outcome AND Limits, Study designs**

| [**# ▲**](http://ovidsp.uk.ovid.com/sp-3.8.1a/ovidweb.cgi?&S=DCDLPDGALHHFOEDMFNOKEBEGNLKJAA00&Sort+Sets=descending) | | **Searches** | **Results** | |
| --- | --- | --- | --- | --- |
| **Population** | | | | |
|  | geriatrics.mp. or exp geriatrics/ | | | 30457 |
|  | geriatric patient.mp. | | | 1211 |
|  | geriatric*.mp. | | | 77878 |
|  | (elder$ or geriatric$).ab,ti. | | | 202883 |
|  | elder*.mp. | | | 186232 |
|  | frail elderly.mp. or exp frail elderly/ | | | 7653 |
|  | aged.mp. or exp Aged/ | | | 4071835 |
|  | old*.mp. | | | 978191 |
|  | old* adult*.mp. | | | 38551 |
|  | old* people*.mp. | | | 18128 |
|  | >65.mp. | | | 283298 |
|  | over 65.mp. | | | 5094 |
|  | or/1-12 | | | 4798174 |
| **Condition** | | | | |
|  | high blood pressure.mp. | | | 10622 |
|  | hypertension.mp. or exp hypertension/ | | | 368517 |
|  | or/14-15 | | | 371621 |
| **Intervention** | | | | |
|  | beta-blocker.mp. | | | 9653 |
|  | beta blocking agent*.mp. | | | 1799 |
|  | Adrenergic beta-Antagonists.mp. or exp Adrenergic beta-Antagonists/ | | | 79060 |
|  | alprenolol.mp. | | | 1563 |
|  | oxprenolol.mp. | | | 1319 |
|  | pindolol.mp. | | | 4644 |
|  | propranolol.mp. | | | 42645 |
|  | timolol.mp. | | | 4297 |
|  | sotalol.mp. | | | 2923 |
|  | nadolol.mp. | | | 1231 |
|  | mepindolol.mp. | | | 86 |
|  | carteolol.mp. | | | 432 |
|  | tertatolol.mp. | | | 178 |
|  | bopindolol.mp. | | | 137 |
|  | bupranolol.mp. | | | 359 |
|  | penbutolol.mp. | | | 258 |
|  | cloranolol.mp. | | | 2 |
|  | practolol.mp. | | | 2117 |
|  | metoprolol.mp. | | | 7219 |
|  | atenolol.mp. | | | 7453 |
|  | acebutolol.mp. | | | 1068 |
|  | betaxolol.mp. | | | 914 |
|  | bevantolol.mp. | | | 82 |
|  | bisoprolol.mp. | | | 1353 |
|  | celiprolol.mp. | | | 504 |
|  | esmolol.mp. | | | 1113 |
|  | epanolol.mp. | | | 60 |
|  | s-atenolol.mp. | | | 39 |
|  | nebivolol.mp. | | | 785 |
|  | talinolol.mp. | | | 240 |
|  | labetalol.mp. | | | 2232 |
|  | carvedilol.mp. | | | 4630 |
|  | or/17-48 | | | 96733 |
| **Outcome** | | | | |
|  | mortality.mp. or exp mortality/ | | | 659917 |
|  | quality of life.mp. or exp quality of life/ | | | 197052 |
|  | QOL.mp. | | | 19540 |
|  | cardiovascular event.mp. | | | 2192 |
|  | myocardial infarction.mp. | | | 190015 |
|  | stroke.mp. | | | 179151 |
|  | hospitalization.mp. or exp hospitalization/ | | | 207457 |
|  | hospitalisation.mp. or exp hospitalisation/ | | | 9370 |
|  | life expectancy.mp. | | | 28007 |
|  | cognitive impairment.mp. | | | 28230 |
|  | cognitive status.mp. | | | 2922 |
|  | functional status.mp. | | | 17274 |
|  | functional impairment.mp. | | | 10027 |
|  | renal failure.mp. | | | 74542 |
|  | renal insufficiency.mp. or exp renal insufficiency/ | | | 133253 |
|  | adverse drug event.mp. | | | 437 |
|  | adverse effects.mp. or exp adverse effects/ | | | 85435 |
|  | drug toxicity.mp. or exp drug toxicity/ | | | 43875 |
|  | safety.mp. | | | 323553 |
|  | patient safety.mp. or exp patient safety/ | | | 16206 |
|  | falls.mp. | | | 36982 |
|  | delirium.mp. or exp delirium/ | | | 19002 |
|  | or/50-71 | | | 1886497 |
| **Limits, Study designs** | | | | |
|  | (systematic review.ti. or meta-analysis.pt. or meta-analysis.ti. or systematic literature review.ti. or (systematic review.ti,ab. and review.pt.) or consensus development conference.pt. or practice guideline.pt. or cochrane database of systematic reviews.jn. or acp journal club.jn. or health technology assessment winchester england.jn. or evidence report technology assessment summary.jn. or drug class reviews.ti. or (clinical guideline and management).tw. or ((evidence based.ti. or evidence-based medicine.sh. or best practice*.ti. or evidence synthesis.ti,ab.) and (((review.pt. or diseases category.mp. or behaviour.sh.) and behavior mechanisms.mp.) or therapeutics.sh. or evaluation studies.pt. or validation studies.pt. or guideline.pt. or pmcbook.mp.)) or (((systematic or systematically).tw. or critical.ti,ab. or study selection.tw. or ((predetermined or inclusion) and criteri*).tw. or exclusion criteri*.tw. or main outcome measures.tw. or standard of care.tw. or standards of care.tw.) and ((survey or surveys).ti,ab. or overview*.tw. or review.ti,ab. or reviews.ti,ab. or search*.tw. or handsearch.tw. or analysis.ti,ab. or critique.ti,ab. or appraisal.tw. or (reduction.tw. and (risk.sh. or risk.tw.) and (death or recurrence).mp.)) and ((literature or articles or publications or publication or bibliography or bibliographies or published).ti,ab. or unpublished.tw. or citation.tw. or citations.tw. or database.ti,ab. or internet.ti,ab. or textbooks.ti,ab. or references.tw. or scales.tw. or papers.tw. or datasets.tw. or trials.ti,ab. or meta-analy*.tw. or (clinical and studies).ti,ab. or treatment outcome.sh. or treatment outcome.tw. or pmcbook.mp.))) not (letter or newspaper article or comment).pt. | | | 205357 |
| **Population AND Condition AND Intervention AND Outcome AND Limits, Study designs** | | | | |
|  | 13 and 16 and 49 and 72 and 73 | | | 97 |

**No** **Limits**

**Total hits: =97**

**No. of duplicates = 16**

**International Pharmaceutical Abstracts 1970 to October 2013 (17.10.2013)**

**No results**

**EBM Reviews - Health Technology Assessment 3rd Quarter 2013**

**(17.10.2013)**

**No results**

**UPDATES (22nd December 2015)**

**1st & 2^nd^ search updates: Beta-Blocking Agents in the management of Hypertension**

**Population AND Condition AND Intervention AND Outcome AND Limits, Study designs**

**Ovid MEDLINE(R) 1946 to November Week 3 2015, Database Field Guide Ovid MEDLINE(R) In-Process & Other Non-Indexed Citations December 21, 2015**

**(22.12.2015)**

| # ▲ | Searches | Results |
| --- | --- | --- |
| 1 | geriatrics.mp. or exp geriatrics/ | 30800 |
| 2 | geriatric patient.mp. | 1281 |
| 3 | geriatric*.mp. | 83491 |
| 4 | (elder$ or geriatric$).ab,ti. | 223965 |
| 5 | elder*.mp. | 207801 |
| 6 | frail elderly.mp. or exp frail elderly/ | 8900 |
| 7 | aged.mp. or exp Aged/ | 4430222 |
| 8 | old*.mp. | 1103472 |
| 9 | old* adult*.mp. | 47776 |
| 10 | old* people*.mp. | 21733 |
| 11 | >65.mp. | 313993 |
| 12 | over 65.mp. | 5723 |
| 13 | or/1-12 | 5250264 |
| 14 | hypertension.mp. or exp hypertension/ | 402215 |
| 15 | high blood pressure.mp. | 11769 |
| 16 | or/14-15 | 405798 |
| 17 | beta-blocker.mp. | 10711 |
| 18 | beta blocking agent*.mp. | 1786 |
| 19 | Adrenergic beta-Antagonists.mp. or exp Adrenergic beta-Antagonists/ | 80021 |
| 20 | alprenolol.mp. | 1572 |
| 21 | oxprenolol.mp. | 1349 |
| 22 | pindolol.mp. | 4663 |
| 23 | propranolol.mp. | 43408 |
| 24 | timolol.mp. | 4672 |
| 25 | sotalol.mp. | 3005 |
| 26 | nadolol.mp. | 1248 |
| 27 | mepindolol.mp. | 86 |
| 28 | carteolol.mp. | 462 |
| 29 | tertatolol.mp. | 175 |
| 30 | bopindolol.mp. | 136 |
| 31 | bupranolol.mp. | 341 |
| 32 | penbutolol.mp. | 261 |
| 33 | cloranolol.mp. | 2 |
| 34 | practolol.mp. | 2170 |
| 35 | metoprolol.mp. | 7310 |
| 36 | atenolol.mp. | 7701 |
| 37 | acebutolol.mp. | 1084 |
| 38 | betaxolol.mp. | 975 |
| 39 | bevantolol.mp. | 81 |
| 40 | bisoprolol.mp. | 1360 |
| 41 | celiprolol.mp. | 497 |
| 42 | esmolol.mp. | 1198 |
| 43 | epanolol.mp. | 58 |
| 44 | s-atenolol.mp. | 39 |
| 45 | nebivolol.mp. | 851 |
| 46 | talinolol.mp. | 242 |
| 47 | labetalol.mp. | 2334 |
| 48 | carvedilol.mp. | 2965 |
| 49 | or/17-48 | 98869 |
| 50 | mortality.mp. or exp mortality/ | 744643 |
| 51 | quality of life.mp. or exp quality of life/ | 230406 |
| 52 | QOL.mp. | 23795 |
| 53 | cardiovascular event.mp. | 2708 |
| 54 | myocardial infarction.mp. | 205589 |
| 55 | stroke.mp. | 209175 |
| 56 | hospitalization.mp. or exp hospitalization/ | 235168 |
| 57 | hospitalisation.mp. or exp hospitalisation/ | 10547 |
| 58 | life expectancy.mp. | 31303 |
| 59 | cognitive impairment.mp. | 36033 |
| 60 | cognitive status.mp. | 3490 |
| 61 | functional status.mp. | 19203 |
| 62 | functional impairment.mp. | 11367 |
| 63 | renal failure.mp. | 80832 |
| 64 | renal insufficiency.mp. or exp renal insufficiency/ | 149956 |
| 65 | adverse drug event.mp. | 551 |
| 66 | adverse effects.mp. or exp adverse effects/ | 95367 |
| 67 | drug toxicity.mp. or exp drug toxicity/ | 99378 |
| 68 | safety.mp. | 378297 |
| 69 | patient safety.mp. or exp patient safety/ | 23817 |
| 70 | falls.mp. | 42025 |
| 71 | delirium.mp. or exp delirium/ | 20984 |
| 72 | or/50-71 | 2185170 |
| 73 | (systematic review.ti. or meta-analysis.pt. or meta-analysis.ti. or systematic literature review.ti. or (systematic review.ti,ab. and review.pt.) or consensus development conference.pt. or practice guideline.pt. or cochrane database of systematic reviews.jn. or acp journal club.jn. or health technology assessment winchester england.jn. or evidence report technology assessment summary.jn. or drug class reviews.ti. or (clinical guideline and management).tw. or ((evidence based.ti. or evidence-based medicine.sh. or best practice*.ti. or evidence synthesis.ti,ab.) and (((review.pt. or diseases category.mp. or behaviour.sh.) and behavior mechanisms.mp.) or therapeutics.sh. or evaluation studies.pt. or validation studies.pt. or guideline.pt. or pmcbook.mp.)) or ((systematic or systematically or critical or study selection or ((predetermined or inclusion) and criteri*) or exclusion criteri* or main outcome measures or standard of care or standards of care).tw. and ((survey or surveys or overview* or review or reviews or search* or handsearch or analysis or critique or appraisal).tw. or (reduction.tw. and (risk.sh. or risk.tw.) and (death or recurrence).mp.)) and ((literature or articles or publications or publication or bibliography or bibliographies or published or unpublished or citation or citations or database or internet or textbooks or references or scales or papers or datasets or trials or meta-analy* or (clinical and studies)).tw. or treatment outcome.sh. or treatment outcome.tw. or pmcbook.mp.))) not (letter or newspaper article or comment).pt. | 247001 |
| 74 | 13 and 16 and 49 and 72 and 73 | 92 |
| 75 | limit 74 to ed=20131017-20151222 | 9 |

**Results = 9**

**EBM Reviews - Health Technology Assessment 4th Quarter 2015**

**(22.12.2015)**

| | # ▲ | Searches | Results | | --- | --- | --- | | 1 | geriatrics.mp. or exp geriatrics/ | 7 | | 2 | geriatric patient.mp. | 0 | | 3 | geriatric*.mp. | 36 | | 4 | (elder$ or geriatric$).ab,ti. | 64 | | 5 | elder*.mp. | 145 | | 6 | frail elderly.mp. or exp frail elderly/ | 17 | | 7 | aged.mp. or exp Aged/ | 432 | | 8 | old*.mp. | 438 | | 9 | old* adult*.mp. | 33 | | 10 | old* people*.mp. | 51 | | 11 | >65.mp. | 90 | | 12 | over 65.mp. | 6 | | 13 | or/1-12 | 847 | | 14 | hypertension.mp. or exp hypertension/ | 174 | | 15 | high blood pressure.mp. | 19 | | 16 | or/14-15 | 184 | | 17 | beta-blocker.mp. | 5 | | 18 | beta blocking agent*.mp. | 0 | | 19 | Adrenergic beta-Antagonists.mp. or exp Adrenergic beta-Antagonists/ | 6 | | 20 | alprenolol.mp. | 0 | | 21 | oxprenolol.mp. | 0 | | 22 | pindolol.mp. | 0 | | 23 | propranolol.mp. | 2 | | 24 | timolol.mp. | 1 | | 25 | sotalol.mp. | 1 | | 26 | nadolol.mp. | 0 | | 27 | mepindolol.mp. | 0 | | 28 | carteolol.mp. | 0 | | 29 | tertatolol.mp. | 0 | | 30 | bopindolol.mp. | 0 | | 31 | bupranolol.mp. | 0 | | 32 | penbutolol.mp. | 0 | | 33 | cloranolol.mp. | 0 | | 34 | practolol.mp. | 0 | | 35 | metoprolol.mp. | 0 | | 36 | atenolol.mp. | 1 | | 37 | acebutolol.mp. | 0 | | 38 | betaxolol.mp. | 0 | | 39 | bevantolol.mp. | 0 | | 40 | bisoprolol.mp. | 0 | | 41 | celiprolol.mp. | 0 | | 42 | esmolol.mp. | 0 | | 43 | epanolol.mp. | 0 | | 44 | s-atenolol.mp. | 0 | | 45 | nebivolol.mp. | 0 | | 46 | talinolol.mp. | 0 | | 47 | labetalol.mp. | 1 | | 48 | carvedilol.mp. | 1 | | 49 | or/17-48 | 14 | | 50 | mortality.mp. or exp mortality/ | 562 | | 51 | quality of life.mp. or exp quality of life/ | 647 | | 52 | QOL.mp. | 47 | | 53 | cardiovascular event.mp. | 6 | | 54 | myocardial infarction.mp. | 146 | | 55 | stroke.mp. | 296 | | 56 | hospitalization.mp. or exp hospitalization/ | 189 | | 57 | hospitalisation.mp. or exp hospitalisation/ | 29 | | 58 | life expectancy.mp. | 36 | | 59 | cognitive impairment.mp. | 30 | | 60 | cognitive status.mp. | 2 | | 61 | functional status.mp. | 23 | | 62 | functional impairment.mp. | 9 | | 63 | renal failure.mp. | 34 | | 64 | renal insufficiency.mp. or exp renal insufficiency/ | 71 | | 65 | adverse drug event.mp. | 0 | | 66 | adverse effects.mp. or exp adverse effects/ | 202 | | 67 | drug toxicity.mp. or exp drug toxicity/ | 9 | | 68 | safety.mp. | 1232 | | 69 | patient safety.mp. or exp patient safety/ | 64 | | 70 | falls.mp. | 50 | | 71 | delirium.mp. or exp delirium/ | 12 | | 72 | or/50-71 | 2956 | | 73 | (systematic review.ti. or meta-analysis.pt. or meta-analysis.ti. or systematic literature review.ti. or (systematic review.ti,ab. and review.pt.) or consensus development conference.pt. or practice guideline.pt. or cochrane database of systematic reviews.jn. or acp journal club.jn. or health technology assessment winchester england.jn. or evidence report technology assessment summary.jn. or drug class reviews.ti. or (clinical guideline and management).tw. or ((evidence based.ti. or evidence-based medicine.sh. or best practice*.ti. or evidence synthesis.ti,ab.) and (((review.pt. or diseases category.mp. or behaviour.sh.) and behavior mechanisms.mp.) or therapeutics.sh. or evaluation studies.pt. or validation studies.pt. or guideline.pt. or pmcbook.mp.)) or ((systematic or systematically or critical or study selection or ((predetermined or inclusion) and criteri*) or exclusion criteri* or main outcome measures or standard of care or standards of care).tw. and ((survey or surveys or overview* or review or reviews or search* or handsearch or analysis or critique or appraisal).tw. or (reduction.tw. and (risk.sh. or risk.tw.) and (death or recurrence).mp.)) and ((literature or articles or publications or publication or bibliography or bibliographies or published or unpublished or citation or citations or database or internet or textbooks or references or scales or papers or datasets or trials or meta-analy* or (clinical and studies)).tw. or treatment outcome.sh. or treatment outcome.tw. or pmcbook.mp.))) not (letter or newspaper article or comment).pt. | 1901 | | 74 | 13 and 16 and 49 and 72 and 73 | 0 | |  |
| --- | --- | --- | --- | --- | --- | --- | --- | --- | --- | --- | --- | --- | --- | --- | --- | --- | --- | --- | --- | --- | --- | --- | --- | --- | --- | --- | --- | --- | --- | --- | --- | --- | --- | --- | --- | --- | --- | --- | --- | --- | --- | --- | --- | --- | --- | --- | --- | --- | --- | --- | --- | --- | --- | --- | --- | --- | --- | --- | --- | --- | --- | --- | --- | --- | --- | --- | --- | --- | --- | --- | --- | --- | --- | --- | --- | --- | --- | --- | --- | --- | --- | --- | --- | --- | --- | --- | --- | --- | --- | --- | --- | --- | --- | --- | --- | --- | --- | --- | --- | --- | --- | --- | --- | --- | --- | --- | --- | --- | --- | --- | --- | --- | --- | --- | --- | --- | --- | --- | --- | --- | --- | --- | --- | --- | --- | --- | --- | --- | --- | --- | --- | --- | --- | --- | --- | --- | --- | --- | --- | --- | --- | --- | --- | --- | --- | --- | --- | --- | --- | --- | --- | --- | --- | --- | --- | --- | --- | --- | --- | --- | --- | --- | --- | --- | --- | --- | --- | --- | --- | --- | --- | --- | --- | --- | --- | --- | --- | --- | --- | --- | --- | --- | --- | --- | --- | --- | --- | --- | --- | --- | --- | --- | --- | --- | --- | --- | --- | --- | --- | --- | --- | --- | --- | --- | --- | --- | --- | --- | --- | --- | --- | --- | --- | --- | --- | --- | --- | --- | --- | --- | --- | --- | --- | --- | --- | --- |

Bottom of Form

Top of Form

**Results=0**

**EBM Reviews - Cochrane Database of Systematic Reviews 2005 to December 2015**

**(22.12.2015)**

| | [# ▲](http://ovidsp.uk.ovid.com/sp-3.18.0b/ovidweb.cgi?&S=CKHKPDGGMMHFNIINFNJKOFBGCPAOAA00&Sort+Sets=descending) | **Searches** | **Results** | | --- | --- | --- | | 1 | geriatrics.mp. or exp geriatrics/ | 61 | | 2 | geriatric patient.mp. | 1 | | 3 | geriatric*.mp. | 253 | | 4 | (elder$ or geriatric$).ab,ti. | 100 | | 5 | elder*.mp. | 1084 | | 6 | frail elderly.mp. or exp frail elderly/ | 45 | | 7 | aged.mp. or exp Aged/ | 2892 | | 8 | old*.mp. | 4221 | | 9 | old* adult*.mp. | 311 | | 10 | old* people*.mp. | 455 | | 11 | >65.mp. | 2408 | | 12 | over 65.mp. | 2408 | | 13 | or/1-12 | 5941 | | 14 | hypertension.mp. or exp hypertension/ | 1463 | | 15 | high blood pressure.mp. | 286 | | 16 | or/14-15 | 1500 | | 17 | beta-blocker.mp. | 114 | | 18 | beta blocking agent*.mp. | 13 | | 19 | Adrenergic beta-Antagonists.mp. or exp Adrenergic beta-Antagonists/ | 58 | | 20 | alprenolol.mp. | 18 | | 21 | oxprenolol.mp. | 32 | | 22 | pindolol.mp. | 36 | | 23 | propranolol.mp. | 96 | | 24 | timolol.mp. | 43 | | 25 | sotalol.mp. | 33 | | 26 | nadolol.mp. | 34 | | 27 | mepindolol.mp. | 13 | | 28 | carteolol.mp. | 22 | | 29 | tertatolol.mp. | 11 | | 30 | bopindolol.mp. | 12 | | 31 | bupranolol.mp. | 16 | | 32 | penbutolol.mp. | 17 | | 33 | cloranolol.mp. | 9 | | 34 | practolol.mp. | 18 | | 35 | metoprolol.mp. | 64 | | 36 | atenolol.mp. | 62 | | 37 | acebutolol.mp. | 28 | | 38 | betaxolol.mp. | 23 | | 39 | bevantolol.mp. | 11 | | 40 | bisoprolol.mp. | 31 | | 41 | celiprolol.mp. | 23 | | 42 | esmolol.mp. | 27 | | 43 | epanolol.mp. | 12 | | 44 | s-atenolol.mp. | 0 | | 45 | nebivolol.mp. | 19 | | 46 | talinolol.mp. | 11 | | 47 | labetalol.mp. | 36 | | 48 | carvedilol.mp. | 37 | | 49 | or/17-48 | 214 | | 50 | mortality.mp. or exp mortality/ | 4417 | | 51 | quality of life.mp. or exp quality of life/ | 4656 | | 52 | QOL.mp. | 692 | | 53 | cardiovascular event.mp. | 92 | | 54 | myocardial infarction.mp. | 775 | | 55 | stroke.mp. | 1263 | | 56 | hospitalization.mp. or exp hospitalization/ | 522 | | 57 | hospitalisation.mp. or exp hospitalisation/ | 1618 | | 58 | life expectancy.mp. | 322 | | 59 | cognitive impairment.mp. | 434 | | 60 | cognitive status.mp. | 54 | | 61 | functional status.mp. | 393 | | 62 | functional impairment.mp. | 173 | | 63 | renal failure.mp. | 542 | | 64 | renal insufficiency.mp. or exp renal insufficiency/ | 145 | | 65 | adverse drug event.mp. | 7 | | 66 | adverse effects.mp. or exp adverse effects/ | 5446 | | 67 | drug toxicity.mp. or exp drug toxicity/ | 81 | | 68 | safety.mp. | 4116 | | 69 | patient safety.mp. or exp patient safety/ | 117 | | 70 | falls.mp. | 511 | | 71 | delirium.mp. or exp delirium/ | 143 | | 72 | or/50-71 | 8373 | | 73 | (systematic review.ti. or meta-analysis.pt. or meta-analysis.ti. or systematic literature review.ti. or (systematic review.ti,ab. and review.pt.) or consensus development conference.pt. or practice guideline.pt. or cochrane database of systematic reviews.jn. or acp journal club.jn. or health technology assessment winchester england.jn. or evidence report technology assessment summary.jn. or drug class reviews.ti. or (clinical guideline and management).tw. or ((evidence based.ti. or evidence-based medicine.sh. or best practice*.ti. or evidence synthesis.ti,ab.) and (((review.pt. or diseases category.mp. or behaviour.sh.) and behavior mechanisms.mp.) or therapeutics.sh. or evaluation studies.pt. or validation studies.pt. or guideline.pt. or pmcbook.mp.)) or (((systematic or systematically).tw. or critical.ti,ab. or study selection.tw. or ((predetermined or inclusion) and criteri*).tw. or exclusion criteri*.tw. or main outcome measures.tw. or standard of care.tw. or standards of care.tw.) and ((survey or surveys).ti,ab. or overview*.tw. or review.ti,ab. or reviews.ti,ab. or search*.tw. or handsearch.tw. or analysis.ti,ab. or critique.ti,ab. or appraisal.tw. or (reduction.tw. and (risk.sh. or risk.tw.) and (death or recurrence).mp.)) and ((literature or articles or publications or publication or bibliography or bibliographies or published).ti,ab. or unpublished.tw. or citation.tw. or citations.tw. or database.ti,ab. or internet.ti,ab. or textbooks.ti,ab. or references.tw. or scales.tw. or papers.tw. or datasets.tw. or trials.ti,ab. or meta-analy*.tw. or (clinical and studies).ti,ab. or treatment outcome.sh. or treatment outcome.tw. or pmcbook.mp.))) not (letter or newspaper article or comment).pt. | 9696 | | 74 | 13 and 16 and 49 and 72 and 73 | 99 | | **75** | **limit 74 to last 3 years** | **50** | |  |
| --- | --- | --- | --- | --- | --- | --- | --- | --- | --- | --- | --- | --- | --- | --- | --- | --- | --- | --- | --- | --- | --- | --- | --- | --- | --- | --- | --- | --- | --- | --- | --- | --- | --- | --- | --- | --- | --- | --- | --- | --- | --- | --- | --- | --- | --- | --- | --- | --- | --- | --- | --- | --- | --- | --- | --- | --- | --- | --- | --- | --- | --- | --- | --- | --- | --- | --- | --- | --- | --- | --- | --- | --- | --- | --- | --- | --- | --- | --- | --- | --- | --- | --- | --- | --- | --- | --- | --- | --- | --- | --- | --- | --- | --- | --- | --- | --- | --- | --- | --- | --- | --- | --- | --- | --- | --- | --- | --- | --- | --- | --- | --- | --- | --- | --- | --- | --- | --- | --- | --- | --- | --- | --- | --- | --- | --- | --- | --- | --- | --- | --- | --- | --- | --- | --- | --- | --- | --- | --- | --- | --- | --- | --- | --- | --- | --- | --- | --- | --- | --- | --- | --- | --- | --- | --- | --- | --- | --- | --- | --- | --- | --- | --- | --- | --- | --- | --- | --- | --- | --- | --- | --- | --- | --- | --- | --- | --- | --- | --- | --- | --- | --- | --- | --- | --- | --- | --- | --- | --- | --- | --- | --- | --- | --- | --- | --- | --- | --- | --- | --- | --- | --- | --- | --- | --- | --- | --- | --- | --- | --- | --- | --- | --- | --- | --- | --- | --- | --- | --- | --- | --- | --- | --- | --- | --- | --- | --- | --- | --- | --- |

Results = 50

**EBM Reviews - Database of Abstracts of Reviews of Effects 2nd Quarter 2015 (22**.12.2015)

| | [# ▲](http://ovidsp.uk.ovid.com/sp-3.18.0b/ovidweb.cgi?&S=CKHKPDGGMMHFNIINFNJKOFBGCPAOAA00&Sort+Sets=descending) | **Searches** | **Results** | | --- | --- | --- | | 1 | geriatrics.mp. or exp geriatrics/ | 41 | | 2 | geriatric patient.mp. | 2 | | 3 | geriatric*.mp. | 297 | | 4 | (elder$ or geriatric$).ab,ti. | 280 | | 5 | elder*.mp. | 722 | | 6 | frail elderly.mp. or exp frail elderly/ | 65 | | 7 | aged.mp. or exp Aged/ | 5512 | | 8 | old*.mp. | 1988 | | 9 | old* adult*.mp. | 340 | | 10 | old* people*.mp. | 198 | | 11 | >65.mp. | 860 | | 12 | over 65.mp. | 66 | | 13 | or/1-12 | 6916 | | 14 | hypertension.mp. or exp hypertension/ | 1163 | | 15 | high blood pressure.mp. | 30 | | 16 | or/14-15 | 1175 | | 17 | beta-blocker.mp. | 95 | | 18 | beta blocking agent*.mp. | 8 | | 19 | Adrenergic beta-Antagonists.mp. or exp Adrenergic beta-Antagonists/ | 179 | | 20 | alprenolol.mp. | 2 | | 21 | oxprenolol.mp. | 15 | | 22 | pindolol.mp. | 27 | | 23 | propranolol.mp. | 75 | | 24 | timolol.mp. | 39 | | 25 | sotalol.mp. | 34 | | 26 | nadolol.mp. | 16 | | 27 | mepindolol.mp. | 0 | | 28 | carteolol.mp. | 1 | | 29 | tertatolol.mp. | 0 | | 30 | bopindolol.mp. | 2 | | 31 | bupranolol.mp. | 1 | | 32 | penbutolol.mp. | 2 | | 33 | cloranolol.mp. | 0 | | 34 | practolol.mp. | 5 | | 35 | metoprolol.mp. | 77 | | 36 | atenolol.mp. | 80 | | 37 | acebutolol.mp. | 14 | | 38 | betaxolol.mp. | 9 | | 39 | bevantolol.mp. | 0 | | 40 | bisoprolol.mp. | 39 | | 41 | celiprolol.mp. | 5 | | 42 | esmolol.mp. | 10 | | 43 | epanolol.mp. | 3 | | 44 | s-atenolol.mp. | 0 | | 45 | nebivolol.mp. | 15 | | 46 | talinolol.mp. | 0 | | 47 | labetalol.mp. | 18 | | 48 | carvedilol.mp. | 43 | | 49 | or/17-48 | 370 | | 50 | mortality.mp. or exp mortality/ | 4529 | | 51 | quality of life.mp. or exp quality of life/ | 2213 | | 52 | QOL.mp. | 68 | | 53 | cardiovascular event.mp. | 54 | | 54 | myocardial infarction.mp. | 1272 | | 55 | stroke.mp. | 1501 | | 56 | hospitalization.mp. or exp hospitalization/ | 311 | | 57 | hospitalisation.mp. or exp hospitalisation/ | 515 | | 58 | life expectancy.mp. | 40 | | 59 | cognitive impairment.mp. | 172 | | 60 | cognitive status.mp. | 15 | | 61 | functional status.mp. | 204 | | 62 | functional impairment.mp. | 30 | | 63 | renal failure.mp. | 179 | | 64 | renal insufficiency.mp. or exp renal insufficiency/ | 161 | | 65 | adverse drug event.mp. | 1 | | 66 | adverse effects.mp. or exp adverse effects/ | 6145 | | 67 | drug toxicity.mp. or exp drug toxicity/ | 34 | | 68 | safety.mp. | 3479 | | 69 | patient safety.mp. or exp patient safety/ | 115 | | 70 | falls.mp. | 215 | | 71 | delirium.mp. or exp delirium/ | 86 | | 72 | or/50-71 | 13583 | | 73 | (systematic review.ti. or meta-analysis.pt. or meta-analysis.ti. or systematic literature review.ti. or (systematic review.ti,ab. and review.pt.) or consensus development conference.pt. or practice guideline.pt. or cochrane database of systematic reviews.jn. or acp journal club.jn. or health technology assessment winchester england.jn. or evidence report technology assessment summary.jn. or drug class reviews.ti. or (clinical guideline and management).tw. or ((evidence based.ti. or evidence-based medicine.sh. or best practice*.ti. or evidence synthesis.ti,ab.) and (((review.pt. or diseases category.mp. or behaviour.sh.) and behavior mechanisms.mp.) or therapeutics.sh. or evaluation studies.pt. or validation studies.pt. or guideline.pt. or pmcbook.mp.)) or (((systematic or systematically).tw. or critical.ti,ab. or study selection.tw. or ((predetermined or inclusion) and criteri*).tw. or exclusion criteri*.tw. or main outcome measures.tw. or standard of care.tw. or standards of care.tw.) and ((survey or surveys).ti,ab. or overview*.tw. or review.ti,ab. or reviews.ti,ab. or search*.tw. or handsearch.tw. or analysis.ti,ab. or critique.ti,ab. or appraisal.tw. or (reduction.tw. and (risk.sh. or risk.tw.) and (death or recurrence).mp.)) and ((literature or articles or publications or publication or bibliography or bibliographies or published).ti,ab. or unpublished.tw. or citation.tw. or citations.tw. or database.ti,ab. or internet.ti,ab. or textbooks.ti,ab. or references.tw. or scales.tw. or papers.tw. or datasets.tw. or trials.ti,ab. or meta-analy*.tw. or (clinical and studies).ti,ab. or treatment outcome.sh. or treatment outcome.tw. or pmcbook.mp.))) not (letter or newspaper article or comment).pt. | 32674 | | **74** | **13 and 16 and 49 and 72 and 73** | **53** | |  |
| --- | --- | --- | --- | --- | --- | --- | --- | --- | --- | --- | --- | --- | --- | --- | --- | --- | --- | --- | --- | --- | --- | --- | --- | --- | --- | --- | --- | --- | --- | --- | --- | --- | --- | --- | --- | --- | --- | --- | --- | --- | --- | --- | --- | --- | --- | --- | --- | --- | --- | --- | --- | --- | --- | --- | --- | --- | --- | --- | --- | --- | --- | --- | --- | --- | --- | --- | --- | --- | --- | --- | --- | --- | --- | --- | --- | --- | --- | --- | --- | --- | --- | --- | --- | --- | --- | --- | --- | --- | --- | --- | --- | --- | --- | --- | --- | --- | --- | --- | --- | --- | --- | --- | --- | --- | --- | --- | --- | --- | --- | --- | --- | --- | --- | --- | --- | --- | --- | --- | --- | --- | --- | --- | --- | --- | --- | --- | --- | --- | --- | --- | --- | --- | --- | --- | --- | --- | --- | --- | --- | --- | --- | --- | --- | --- | --- | --- | --- | --- | --- | --- | --- | --- | --- | --- | --- | --- | --- | --- | --- | --- | --- | --- | --- | --- | --- | --- | --- | --- | --- | --- | --- | --- | --- | --- | --- | --- | --- | --- | --- | --- | --- | --- | --- | --- | --- | --- | --- | --- | --- | --- | --- | --- | --- | --- | --- | --- | --- | --- | --- | --- | --- | --- | --- | --- | --- | --- | --- | --- | --- | --- | --- | --- | --- | --- | --- | --- | --- | --- | --- | --- | --- | --- | --- | --- | --- | --- |

Results = 53

IT IS NOT POSSIBLE TO LIMIT THE YEAR OF PUBLICATION IN DARE BECAUSE THE DATABASE IS UPDATED EVERY YEAR, SO ONLY THE LATEST YEAR IS SHOWN. HENCE LIMITING TO THE LAST YEAR SHOWS EXACTLY THE SAME RESULTS AS DEMONSTRATED ABOVE

**International Pharmaceutical Abstracts 1970 to December 2015**

**(22**.12.2015)

| | [# ▲](http://ovidsp.uk.ovid.com/sp-3.18.0b/ovidweb.cgi?&S=CKHKPDGGMMHFNIINFNJKOFBGCPAOAA00&Sort+Sets=descending) | **Searches** | **Results** | | --- | --- | --- | | 1 | geriatrics.mp. or exp geriatrics/ | 10608 | | 2 | geriatric patient.mp. | 146 | | 3 | geriatric*.mp. | 10936 | | 4 | (elder$ or geriatric$).ab,ti. | 9756 | | 5 | elder*.mp. | 8490 | | 6 | frail elderly.mp. or exp frail elderly/ | 73 | | 7 | aged.mp. or exp Aged/ | 16364 | | 8 | old*.mp. | 34739 | | 9 | old* adult*.mp. | 830 | | 10 | old* people*.mp. | 392 | | 11 | >65.mp. | 8978 | | 12 | over 65.mp. | 177 | | 13 | or/1-12 | 62675 | | 14 | hypertension.mp. or exp hypertension/ | 14082 | | 15 | high blood pressure.mp. | 423 | | 16 | or/14-15 | 14161 | | 17 | beta-blocker.mp. | 945 | | 18 | beta blocking agent*.mp. | 132 | | 19 | Adrenergic beta-Antagonists.mp. or exp Adrenergic beta-Antagonists/ | 2 | | 20 | alprenolol.mp. | 126 | | 21 | oxprenolol.mp. | 325 | | 22 | pindolol.mp. | 406 | | 23 | propranolol.mp. | 3366 | | 24 | timolol.mp. | 607 | | 25 | sotalol.mp. | 372 | | 26 | nadolol.mp. | 221 | | 27 | mepindolol.mp. | 26 | | 28 | carteolol.mp. | 66 | | 29 | tertatolol.mp. | 11 | | 30 | bopindolol.mp. | 24 | | 31 | bupranolol.mp. | 23 | | 32 | penbutolol.mp. | 61 | | 33 | cloranolol.mp. | 3 | | 34 | practolol.mp. | 218 | | 35 | metoprolol.mp. | 1490 | | 36 | atenolol.mp. | 1396 | | 37 | acebutolol.mp. | 242 | | 38 | betaxolol.mp. | 108 | | 39 | bevantolol.mp. | 27 | | 40 | bisoprolol.mp. | 224 | | 41 | celiprolol.mp. | 97 | | 42 | esmolol.mp. | 132 | | 43 | epanolol.mp. | 25 | | 44 | s-atenolol.mp. | 6 | | 45 | nebivolol.mp. | 176 | | 46 | talinolol.mp. | 79 | | 47 | labetalol.mp. | 439 | | 48 | carvedilol.mp. | 521 | | 49 | or/17-48 | 9091 | | 50 | mortality.mp. or exp mortality/ | 15319 | | 51 | quality of life.mp. or exp quality of life/ | 7243 | | 52 | QOL.mp. | 574 | | 53 | cardiovascular event.mp. | 183 | | 54 | myocardial infarction.mp. | 6518 | | 55 | stroke.mp. | 4522 | | 56 | hospitalization.mp. or exp hospitalization/ | 4178 | | 57 | hospitalisation.mp. or exp hospitalisation/ | 273 | | 58 | life expectancy.mp. | 574 | | 59 | cognitive impairment.mp. | 489 | | 60 | cognitive status.mp. | 49 | | 61 | functional status.mp. | 272 | | 62 | functional impairment.mp. | 177 | | 63 | renal failure.mp. | 3130 | | 64 | renal insufficiency.mp. or exp renal insufficiency/ | 1094 | | 65 | adverse drug event.mp. | 333 | | 66 | adverse effects.mp. or exp adverse effects/ | 13959 | | 67 | drug toxicity.mp. or exp drug toxicity/ | 486 | | 68 | safety.mp. | 31461 | | 69 | patient safety.mp. or exp patient safety/ | 1751 | | 70 | falls.mp. | 778 | | 71 | delirium.mp. or exp delirium/ | 531 | | 72 | or/50-71 | 76516 | | 73 | (systematic review.ti. or meta-analysis.pt. or meta-analysis.ti. or systematic literature review.ti. or (systematic review.ti,ab. and review.pt.) or consensus development conference.pt. or practice guideline.pt. or cochrane database of systematic reviews.jn. or acp journal club.jn. or health technology assessment winchester england.jn. or evidence report technology assessment summary.jn. or drug class reviews.ti. or (clinical guideline and management).tw. or ((evidence based.ti. or evidence-based medicine.sh. or best practice*.ti. or evidence synthesis.ti,ab.) and (((review.pt. or diseases category.mp. or behaviour.sh.) and behavior mechanisms.mp.) or therapeutics.sh. or evaluation studies.pt. or validation studies.pt. or guideline.pt. or pmcbook.mp.)) or ((systematic or systematically or critical or study selection or ((predetermined or inclusion) and criteri*) or exclusion criteri* or main outcome measures or standard of care or standards of care).tw. and ((survey or surveys or overview* or review or reviews or search* or handsearch or analysis or critique or appraisal).tw. or (reduction.tw. and (risk.sh. or risk.tw.) and (death or recurrence).mp.)) and ((literature or articles or publications or publication or bibliography or bibliographies or published or unpublished or citation or citations or database or internet or textbooks or references or scales or papers or datasets or trials or meta-analy* or (clinical and studies)).tw. or treatment outcome.sh. or treatment outcome.tw. or pmcbook.mp.))) not (letter or newspaper article or comment).pt. | 7965 | | 74 | 13 and 16 and 49 and 72 and 73 | 5 | | **75** | **limit 74 to last 3 years** | **2** | |  |
| --- | --- | --- | --- | --- | --- | --- | --- | --- | --- | --- | --- | --- | --- | --- | --- | --- | --- | --- | --- | --- | --- | --- | --- | --- | --- | --- | --- | --- | --- | --- | --- | --- | --- | --- | --- | --- | --- | --- | --- | --- | --- | --- | --- | --- | --- | --- | --- | --- | --- | --- | --- | --- | --- | --- | --- | --- | --- | --- | --- | --- | --- | --- | --- | --- | --- | --- | --- | --- | --- | --- | --- | --- | --- | --- | --- | --- | --- | --- | --- | --- | --- | --- | --- | --- | --- | --- | --- | --- | --- | --- | --- | --- | --- | --- | --- | --- | --- | --- | --- | --- | --- | --- | --- | --- | --- | --- | --- | --- | --- | --- | --- | --- | --- | --- | --- | --- | --- | --- | --- | --- | --- | --- | --- | --- | --- | --- | --- | --- | --- | --- | --- | --- | --- | --- | --- | --- | --- | --- | --- | --- | --- | --- | --- | --- | --- | --- | --- | --- | --- | --- | --- | --- | --- | --- | --- | --- | --- | --- | --- | --- | --- | --- | --- | --- | --- | --- | --- | --- | --- | --- | --- | --- | --- | --- | --- | --- | --- | --- | --- | --- | --- | --- | --- | --- | --- | --- | --- | --- | --- | --- | --- | --- | --- | --- | --- | --- | --- | --- | --- | --- | --- | --- | --- | --- | --- | --- | --- | --- | --- | --- | --- | --- | --- | --- | --- | --- | --- | --- | --- | --- | --- | --- | --- | --- | --- | --- | --- | --- | --- |

Results = 2

**Embase**1974 to 2015 December 21

(22.12.2015)

| | [# ▲](http://ovidsp.uk.ovid.com/sp-3.18.0b/ovidweb.cgi?&S=CKHKPDGGMMHFNIINFNJKOFBGCPAOAA00&Sort+Sets=descending) | **Searches** | **Results** | | --- | --- | --- | | 1 | geriatrics.mp. or exp geriatrics/ | 51713 | | 2 | geriatric patient.mp. | 18689 | | 3 | geriatric*.mp. | 120401 | | 4 | (elder$ or geriatric$).ab,ti. | 298278 | | 5 | elder*.mp. | 356092 | | 6 | frail elderly.mp. or exp frail elderly/ | 8841 | | 7 | aged.mp. or exp Aged/ | 3280017 | | 8 | old*.mp. | 1404730 | | 9 | old* adult*.mp. | 57649 | | 10 | old* people*.mp. | 26957 | | 11 | >65.mp. | 436232 | | 12 | over 65.mp. | 8500 | | 13 | or/1-12 | 4576282 | | 14 | hypertension.mp. or exp hypertension/ | 697919 | | 15 | high blood pressure.mp. | 15959 | | 16 | or/14-15 | 700063 | | 17 | beta-blocker.mp. | 15610 | | 18 | beta blocking agent*.mp. | 1953 | | 19 | Adrenergic beta-Antagonists.mp. or exp Adrenergic beta-Antagonists/ | 235120 | | 20 | alprenolol.mp. | 3872 | | 21 | oxprenolol.mp. | 4173 | | 22 | pindolol.mp. | 8711 | | 23 | propranolol.mp. | 80336 | | 24 | timolol.mp. | 12525 | | 25 | sotalol.mp. | 11146 | | 26 | nadolol.mp. | 5061 | | 27 | mepindolol.mp. | 346 | | 28 | carteolol.mp. | 1420 | | 29 | tertatolol.mp. | 330 | | 30 | bopindolol.mp. | 294 | | 31 | bupranolol.mp. | 777 | | 32 | penbutolol.mp. | 817 | | 33 | cloranolol.mp. | 45 | | 34 | practolol.mp. | 3075 | | 35 | metoprolol.mp. | 30251 | | 36 | atenolol.mp. | 28148 | | 37 | acebutolol.mp. | 4482 | | 38 | betaxolol.mp. | 3112 | | 39 | bevantolol.mp. | 222 | | 40 | bisoprolol.mp. | 7533 | | 41 | celiprolol.mp. | 1464 | | 42 | esmolol.mp. | 4226 | | 43 | epanolol.mp. | 104 | | 44 | s-atenolol.mp. | 52 | | 45 | nebivolol.mp. | 2992 | | 46 | talinolol.mp. | 608 | | 47 | labetalol.mp. | 9089 | | 48 | carvedilol.mp. | 11944 | | 49 | or/17-48 | 241027 | | 50 | mortality.mp. or exp mortality/ | 1008481 | | 51 | quality of life.mp. or exp quality of life/ | 377629 | | 52 | QOL.mp. | 42640 | | 53 | cardiovascular event.mp. | 4723 | | 54 | myocardial infarction.mp. | 207953 | | 55 | stroke.mp. | 299377 | | 56 | hospitalization.mp. or exp hospitalization/ | 296472 | | 57 | hospitalisation.mp. or exp hospitalisation/ | 17428 | | 58 | life expectancy.mp. | 46453 | | 59 | cognitive impairment.mp. | 55857 | | 60 | cognitive status.mp. | 4982 | | 61 | functional status.mp. | 43981 | | 62 | functional impairment.mp. | 15123 | | 63 | renal failure.mp. | 104184 | | 64 | renal insufficiency.mp. or exp renal insufficiency/ | 253910 | | 65 | adverse drug event.mp. | 966 | | 66 | adverse effects.mp. or exp adverse effects/ | 139484 | | 67 | drug toxicity.mp. or exp drug toxicity/ | 73878 | | 68 | safety.mp. | 747059 | | 69 | patient safety.mp. or exp patient safety/ | 81108 | | 70 | falls.mp. | 42471 | | 71 | delirium.mp. or exp delirium/ | 22751 | | 72 | or/50-71 | 3053853 | | 73 | (systematic review.ti. or meta-analysis.pt. or meta-analysis.ti. or systematic literature review.ti. or (systematic review.ti,ab. and review.pt.) or consensus development conference.pt. or practice guideline.pt. or cochrane database of systematic reviews.jn. or acp journal club.jn. or health technology assessment winchester england.jn. or evidence report technology assessment summary.jn. or drug class reviews.ti. or (clinical guideline and management).tw. or ((evidence based.ti. or evidence-based medicine.sh. or best practice*.ti. or evidence synthesis.ti,ab.) and (((review.pt. or diseases category.mp. or behaviour.sh.) and behavior mechanisms.mp.) or therapeutics.sh. or evaluation studies.pt. or validation studies.pt. or guideline.pt. or pmcbook.mp.)) or ((systematic or systematically or critical or study selection or ((predetermined or inclusion) and criteri*) or exclusion criteri* or main outcome measures or standard of care or standards of care).tw. and ((survey or surveys or overview* or review or reviews or search* or handsearch or analysis or critique or appraisal).tw. or (reduction.tw. and (risk.sh. or risk.tw.) and (death or recurrence).mp.)) and ((literature or articles or publications or publication or bibliography or bibliographies or published or unpublished or citation or citations or database or internet or textbooks or references or scales or papers or datasets or trials or meta-analy* or (clinical and studies)).tw. or treatment outcome.sh. or treatment outcome.tw. or pmcbook.mp.))) not (letter or newspaper article or comment).pt. | 256315 | | 74 | 13 and 16 and 49 and 72 and 73 | 199 | | 75 | limit 74 to dd=20131017-20151222 | 30 | | **76** | **limit 75 to yr="2013-2015"** | **29** | |  |
| --- | --- | --- | --- | --- | --- | --- | --- | --- | --- | --- | --- | --- | --- | --- | --- | --- | --- | --- | --- | --- | --- | --- | --- | --- | --- | --- | --- | --- | --- | --- | --- | --- | --- | --- | --- | --- | --- | --- | --- | --- | --- | --- | --- | --- | --- | --- | --- | --- | --- | --- | --- | --- | --- | --- | --- | --- | --- | --- | --- | --- | --- | --- | --- | --- | --- | --- | --- | --- | --- | --- | --- | --- | --- | --- | --- | --- | --- | --- | --- | --- | --- | --- | --- | --- | --- | --- | --- | --- | --- | --- | --- | --- | --- | --- | --- | --- | --- | --- | --- | --- | --- | --- | --- | --- | --- | --- | --- | --- | --- | --- | --- | --- | --- | --- | --- | --- | --- | --- | --- | --- | --- | --- | --- | --- | --- | --- | --- | --- | --- | --- | --- | --- | --- | --- | --- | --- | --- | --- | --- | --- | --- | --- | --- | --- | --- | --- | --- | --- | --- | --- | --- | --- | --- | --- | --- | --- | --- | --- | --- | --- | --- | --- | --- | --- | --- | --- | --- | --- | --- | --- | --- | --- | --- | --- | --- | --- | --- | --- | --- | --- | --- | --- | --- | --- | --- | --- | --- | --- | --- | --- | --- | --- | --- | --- | --- | --- | --- | --- | --- | --- | --- | --- | --- | --- | --- | --- | --- | --- | --- | --- | --- | --- | --- | --- | --- | --- | --- | --- | --- | --- | --- | --- | --- | --- | --- | --- | --- | --- | --- | --- | --- | --- |

Results=29

**For all databases (Search 1, 2 and 3A):-**

**No of references found = 143**

**No of duplicates = 6**

**Total remaining = 137**

**SEARCH 3B (28/9/2016)**

**Search 3B: Beta-Blocking Agents in the management of Hypertension**

**Population AND Condition AND Intervention AND Outcome AND Limits, Study designs (28/9/2016)**

**Medline**

Ovid MEDLINE(R) 1946 to September Week 2 2016 (28/9/2016)

| 1 | geriatrics.mp. or exp geriatrics/ | 30884 |
| --- | --- | --- |
| 2 | geriatric patient.mp. | 1262 |
| 3 | geriatric*.mp. | 82975 |
| 4 | (elder$ or geriatric$).ab,ti. | 213364 |
| 5 | elder*.mp. | 198011 |
| 6 | frail elderly.mp. or exp frail elderly/ | 9324 |
| 7 | aged.mp. or exp Aged/ | 4504377 |
| 8 | old*.mp. | 1033387 |
| 9 | old* adult*.mp. | 44869 |
| 10 | old* people*.mp. | 20576 |
| 11 | >65.mp. | 298503 |
| 12 | over 65.mp. | 5429 |
| 13 | or/1-12 | 5220042 |
| 14 | hypertension.mp. or exp hypertension/ | 399980 |
| 15 | high blood pressure.mp. | 11537 |
| 16 | or/14-15 | 403159 |
| 17 | beta-blocker.mp. | 10386 |
| 18 | beta blocking agent*.mp. | 1753 |
| 19 | Adrenergic beta-Antagonists.mp. or exp Adrenergic beta-Antagonists/ | 80034 |
| 20 | alprenolol.mp. | 1551 |
| 21 | oxprenolol.mp. | 1315 |
| 22 | pindolol.mp. | 4602 |
| 23 | propranolol.mp. | 42618 |
| 24 | timolol.mp. | 4344 |
| 25 | sotalol.mp. | 2949 |
| 26 | nadolol.mp. | 1217 |
| 27 | mepindolol.mp. | 88 |
| 28 | carteolol.mp. | 426 |
| 29 | tertatolol.mp. | 174 |
| 30 | bopindolol.mp. | 138 |
| 31 | bupranolol.mp. | 330 |
| 32 | penbutolol.mp. | 260 |
| 33 | cloranolol.mp. | 2 |
| 34 | practolol.mp. | 2108 |
| 35 | metoprolol.mp. | 7174 |
| 36 | atenolol.mp. | 7578 |
| 37 | acebutolol.mp. | 1082 |
| 38 | betaxolol.mp. | 918 |
| 39 | bevantolol.mp. | 82 |
| 40 | bisoprolol.mp. | 1347 |
| 41 | celiprolol.mp. | 495 |
| 42 | esmolol.mp. | 1153 |
| 43 | epanolol.mp. | 59 |
| 44 | s-atenolol.mp. | 35 |
| 45 | nebivolol.mp. | 818 |
| 46 | talinolol.mp. | 246 |
| 47 | labetalol.mp. | 2265 |
| 48 | carvedilol.mp. | 2887 |
| 49 | or/17-48 | 96969 |
| 50 | mortality.mp. or exp mortality/ | 724106 |
| 51 | quality of life.mp. or exp quality of life/ | 221149 |
| 52 | QOL.mp. | 22438 |
| 53 | cardiovascular event.mp. | 2622 |
| 54 | myocardial infarction.mp. | 201426 |
| 55 | stroke.mp. | 201353 |
| 56 | hospitalization.mp. or exp hospitalization/ | 238811 |
| 57 | hospitalisation.mp. or exp hospitalisation/ | 10306 |
| 58 | life expectancy.mp. | 30174 |
| 59 | cognitive impairment.mp. | 33734 |
| 60 | cognitive status.mp. | 3297 |
| 61 | functional status.mp. | 18374 |
| 62 | functional impairment.mp. | 10791 |
| 63 | renal failure.mp. | 76207 |
| 64 | renal insufficiency.mp. or exp renal insufficiency/ | 152725 |
| 65 | adverse drug event.mp. | 499 |
| 66 | adverse effects.mp. or exp adverse effects/ | 90664 |
| 67 | drug toxicity.mp. or exp drug toxicity/ | 103173 |
| 68 | safety.mp. | 359822 |
| 69 | patient safety.mp. or exp patient safety/ | 23695 |
| 70 | falls.mp. | 40042 |
| 71 | delirium.mp. or exp delirium/ | 12052 |
| 72 | or/50-71 | 2114204 |
| 73 | randomized controlled trial.pt. | 431071 |
| 74 | controlled clinical trial.pt. | 91705 |
| 75 | randomized.ab. | 327108 |
| 76 | placebo.ab. | 164790 |
| 77 | drug therapy.fs. | 1909098 |
| 78 | randomly.ab. | 229493 |
| 79 | trial.ab. | 340908 |
| 80 | groups.ab. | 1441581 |
| 81 | or/73-80 | 3638642 |
| 82 | exp animals/ not humans.sh. | 4318084 |
| 83 | 81 not 82 | 3102061 |
| 84 | Comparative studies/ | 1768822 |
| 85 | Follow-up studies/ | 561923 |
| 86 | Time factors/ | 1085317 |
| 87 | chang$.tw. | 2276329 |
| 88 | evaluat$.tw. | 2378674 |
| 89 | reviewed.tw. | 367452 |
| 90 | prospective$.tw. | 485118 |
| 91 | retrospective$.tw. | 444461 |
| 92 | baseline.tw. | 373095 |
| 93 | cohort.tw. | 303955 |
| 94 | case series.tw. | 41474 |
| 95 | or/83-94 | 8767237 |
| 96 | 13 and 16 and 49 and 72 and 81 and 95 | 2603 |

Results = 2603

EMBASE

**Search 3B: Beta-Blocking Agents in the management of Hypertension**

**Population AND Condition AND Intervention AND Outcome AND Limits, Study designs (28/9/2016)**

**Embase**1974 to 2016 September 27

| 1 | geriatrics.mp. or exp geriatrics/ | 47763 |
| --- | --- | --- |
| 2 | geriatric patient.mp. | 21459 |
| 3 | geriatric*.mp. | 119907 |
| 4 | (elder$ or geriatric$).ab,ti. | 323466 |
| 5 | elder*.mp. | 389052 |
| 6 | frail elderly.mp. or exp frail elderly/ | 9735 |
| 7 | aged.mp. or exp Aged/ | 3475891 |
| 8 | old*.mp. | 1544917 |
| 9 | old* adult*.mp. | 64201 |
| 10 | old* people*.mp. | 30225 |
| 11 | >65.mp. | 480628 |
| 12 | over 65.mp. | 9385 |
| 13 | or/1-12 | 4878792 |
| 14 | hypertension.mp. or exp hypertension/ | 766633 |
| 15 | high blood pressure.mp. | 17382 |
| 16 | or/14-15 | 768230 |
| 17 | beta-blocker.mp. | 17005 |
| 18 | beta blocking agent*.mp. | 2481 |
| 19 | Adrenergic beta-Antagonists.mp. or exp Adrenergic beta-Antagonists/ | 264470 |
| 20 | alprenolol.mp. | 5304 |
| 21 | oxprenolol.mp. | 5449 |
| 22 | pindolol.mp. | 10487 |
| 23 | propranolol.mp. | 96074 |
| 24 | timolol.mp. | 13482 |
| 25 | sotalol.mp. | 12182 |
| 26 | nadolol.mp. | 5278 |
| 27 | mepindolol.mp. | 375 |
| 28 | carteolol.mp. | 1534 |
| 29 | tertatolol.mp. | 343 |
| 30 | bopindolol.mp. | 297 |
| 31 | bupranolol.mp. | 973 |
| 32 | penbutolol.mp. | 906 |
| 33 | cloranolol.mp. | 54 |
| 34 | practolol.mp. | 5413 |
| 35 | metoprolol.mp. | 32254 |
| 36 | atenolol.mp. | 29524 |
| 37 | acebutolol.mp. | 5033 |
| 38 | betaxolol.mp. | 3217 |
| 39 | bevantolol.mp. | 240 |
| 40 | bisoprolol.mp. | 8024 |
| 41 | celiprolol.mp. | 1486 |
| 42 | esmolol.mp. | 4444 |
| 43 | epanolol.mp. | 106 |
| 44 | s-atenolol.mp. | 53 |
| 45 | nebivolol.mp. | 3193 |
| 46 | talinolol.mp. | 644 |
| 47 | labetalol.mp. | 9721 |
| 48 | carvedilol.mp. | 12717 |
| 49 | or/17-48 | 270260 |
| 50 | mortality.mp. or exp mortality/ | 1107555 |
| 51 | quality of life.mp. or exp quality of life/ | 417666 |
| 52 | QOL.mp. | 48005 |
| 53 | cardiovascular event.mp. | 5245 |
| 54 | myocardial infarction.mp. | 229873 |
| 55 | stroke.mp. | 327958 |
| 56 | hospitalization.mp. or exp hospitalization/ | 342694 |
| 57 | hospitalisation.mp. or exp hospitalisation/ | 19258 |
| 58 | life expectancy.mp. | 50697 |
| 59 | cognitive impairment.mp. | 62206 |
| 60 | cognitive status.mp. | 5465 |
| 61 | functional status.mp. | 52476 |
| 62 | functional impairment.mp. | 16747 |
| 63 | renal failure.mp. | 113644 |
| 64 | renal insufficiency.mp. or exp renal insufficiency/ | 291620 |
| 65 | adverse drug event.mp. | 1058 |
| 66 | adverse effects.mp. or exp adverse effects/ | 152493 |
| 67 | drug toxicity.mp. or exp drug toxicity/ | 576668 |
| 68 | safety.mp. | 819176 |
| 69 | patient safety.mp. or exp patient safety/ | 92437 |
| 70 | falls.mp. | 47678 |
| 71 | delirium.mp. or exp delirium/ | 25250 |
| 72 | or/50-71 | 3793238 |
| 73 | random$.mp. | 1310275 |
| 74 | factorial$.mp. | 65309 |
| 75 | crossover$.mp. | 80843 |
| 76 | cross over$.mp. | 26566 |
| 77 | cross-over$.mp. | 26566 |
| 78 | placebo$.mp. | 384331 |
| 79 | (doubl$ adj blind$).mp. | 213187 |
| 80 | (singl$ adj blind$).mp. | 35241 |
| 81 | assign$.mp. | 299137 |
| 82 | allocat$.mp. | 121959 |
| 83 | volunteer$.mp. | 221003 |
| 84 | crossover procedure/ | 52956 |
| 85 | double blind procedure/ | 135930 |
| 86 | randomized controlled trial/ | 451612 |
| 87 | single blind procedure/ | 25723 |
| 88 | or/73-87 | 2006085 |
| 89 | Controlled study/ | 5205014 |
| 90 | Treatment outcome/ | 730547 |
| 91 | Major clinical study/ | 2632521 |
| 92 | Clinical trial/ | 972910 |
| 93 | chang$.tw. | 3080809 |
| 94 | evaluat$.tw. | 3599197 |
| 95 | reviewed.tw. | 560608 |
| 96 | baseline.tw. | 643012 |
| 97 | (compare$ or compara$).tw. | 4451253 |
| 98 | or/89-97 | 13234748 |
| 99 | 13 and 16 and 49 and 72 and 88 and 98 | 2748 |

Results = 2748

HTA

**EBM Reviews - Health Technology Assessment**3rd Quarter 2016

**Search 3B: Beta-Blocking Agents in the management of Hypertension**

**Population AND Condition AND Intervention AND Outcome AND Limits, Study designs (28/9/2016)**

| 1 | geriatrics.mp. or exp geriatrics/ | 7 |
| --- | --- | --- |
| 2 | geriatric patient.mp. | 0 |
| 3 | geriatric*.mp. | 39 |
| 4 | (elder$ or geriatric$).ab,ti. | 69 |
| 5 | elder*.mp. | 157 |
| 6 | frail elderly.mp. or exp frail elderly/ | 16 |
| 7 | aged.mp. or exp Aged/ | 483 |
| 8 | old*.mp. | 466 |
| 9 | old* adult*.mp. | 35 |
| 10 | old* people*.mp. | 58 |
| 11 | >65.mp. | 97 |
| 12 | over 65.mp. | 10 |
| 13 | or/1-12 | 932 |
| 14 | hypertension.mp. or exp hypertension/ | 186 |
| 15 | high blood pressure.mp. | 20 |
| 16 | or/14-15 | 197 |
| 17 | beta-blocker.mp. | 5 |
| 18 | beta blocking agent*.mp. | 0 |
| 19 | Adrenergic beta-Antagonists.mp. or exp Adrenergic beta-Antagonists/ | 6 |
| 20 | alprenolol.mp. | 0 |
| 21 | oxprenolol.mp. | 0 |
| 22 | pindolol.mp. | 0 |
| 23 | propranolol.mp. | 3 |
| 24 | timolol.mp. | 3 |
| 25 | sotalol.mp. | 1 |
| 26 | nadolol.mp. | 0 |
| 27 | mepindolol.mp. | 0 |
| 28 | carteolol.mp. | 0 |
| 29 | tertatolol.mp. | 0 |
| 30 | bopindolol.mp. | 0 |
| 31 | bupranolol.mp. | 0 |
| 32 | penbutolol.mp. | 0 |
| 33 | cloranolol.mp. | 0 |
| 34 | practolol.mp. | 0 |
| 35 | metoprolol.mp. | 0 |
| 36 | atenolol.mp. | 1 |
| 37 | acebutolol.mp. | 0 |
| 38 | betaxolol.mp. | 0 |
| 39 | bevantolol.mp. | 0 |
| 40 | bisoprolol.mp. | 0 |
| 41 | celiprolol.mp. | 0 |
| 42 | esmolol.mp. | 0 |
| 43 | epanolol.mp. | 0 |
| 44 | s-atenolol.mp. | 0 |
| 45 | nebivolol.mp. | 0 |
| 46 | talinolol.mp. | 0 |
| 47 | labetalol.mp. | 1 |
| 48 | carvedilol.mp. | 1 |
| 49 | or/17-48 | 17 |
| 50 | mortality.mp. or exp mortality/ | 592 |
| 51 | quality of life.mp. or exp quality of life/ | 709 |
| 52 | QOL.mp. | 48 |
| 53 | cardiovascular event.mp. | 6 |
| 54 | myocardial infarction.mp. | 150 |
| 55 | stroke.mp. | 312 |
| 56 | hospitalization.mp. or exp hospitalization/ | 184 |
| 57 | hospitalisation.mp. or exp hospitalisation/ | 35 |
| 58 | life expectancy.mp. | 43 |
| 59 | cognitive impairment.mp. | 31 |
| 60 | cognitive status.mp. | 2 |
| 61 | functional status.mp. | 24 |
| 62 | functional impairment.mp. | 10 |
| 63 | renal failure.mp. | 36 |
| 64 | renal insufficiency.mp. or exp renal insufficiency/ | 76 |
| 65 | adverse drug event.mp. | 0 |
| 66 | adverse effects.mp. or exp adverse effects/ | 211 |
| 67 | drug toxicity.mp. or exp drug toxicity/ | 6 |
| 68 | safety.mp. | 1328 |
| 69 | patient safety.mp. or exp patient safety/ | 65 |
| 70 | falls.mp. | 54 |
| 71 | delirium.mp. or exp delirium/ | 11 |
| 72 | or/50-71 | 3163 |
| 73 | randomized controlled trial.pt. | 0 |
| 74 | controlled clinical trial.pt. | 0 |
| 75 | [randomized.ab.] | 0 |
| 76 | [placebo.ab.] | 0 |
| 77 | drug therapy.fs. | 1165 |
| 78 | [randomly.ab.] | 0 |
| 79 | [trial.ab.] | 0 |
| 80 | [groups.ab.] | 0 |
| 81 | or/73-80 | 1165 |
| 82 | exp animals/ not humans.sh. | 30 |
| 83 | 81 not 82 | 1161 |
| 84 | Comparative studies/ | 39 |
| 85 | Follow-up studies/ | 24 |
| 86 | Time factors/ | 69 |
| 87 | chang$.tw. | 624 |
| 88 | evaluat$.tw. | 14569 |
| 89 | reviewed.tw. | 216 |
| 90 | prospective$.tw. | 210 |
| 91 | retrospective$.tw. | 91 |
| 92 | baseline.tw. | 118 |
| 93 | cohort.tw. | 129 |
| 94 | case series.tw. | 172 |
| 95 | or/83-94 | 14756 |
| 96 | 13 and 16 and 49 and 72 and 81 and 95 | 1 |

Results = 1

IPA

**International Pharmaceutical Abstracts**1970 to September 2016

**Search 3B: Beta-Blocking Agents in the management of Hypertension**

**Population AND Condition AND Intervention AND Outcome AND Limits, Study designs (28/9/2016)**

| 1 | geriatrics.mp. or exp geriatrics/ | 10805 |
| --- | --- | --- |
| 2 | geriatric patient.mp. | 149 |
| 3 | geriatric*.mp. | 11141 |
| 4 | (elder$ or geriatric$).ab,ti. | 9932 |
| 5 | elder*.mp. | 8645 |
| 6 | frail elderly.mp. or exp frail elderly/ | 76 |
| 7 | aged.mp. or exp Aged/ | 16653 |
| 8 | old*.mp. | 35501 |
| 9 | old* adult*.mp. | 899 |
| 10 | old* people*.mp. | 414 |
| 11 | >65.mp. | 9233 |
| 12 | over 65.mp. | 177 |
| 13 | or/1-12 | 63931 |
| 14 | hypertension.mp. or exp hypertension/ | 14354 |
| 15 | high blood pressure.mp. | 435 |
| 16 | or/14-15 | 14438 |
| 17 | beta-blocker.mp. | 960 |
| 18 | beta blocking agent*.mp. | 135 |
| 19 | Adrenergic beta-Antagonists.mp. or exp Adrenergic beta-Antagonists/ | 2 |
| 20 | alprenolol.mp. | 126 |
| 21 | oxprenolol.mp. | 325 |
| 22 | pindolol.mp. | 406 |
| 23 | propranolol.mp. | 3406 |
| 24 | timolol.mp. | 624 |
| 25 | sotalol.mp. | 377 |
| 26 | nadolol.mp. | 221 |
| 27 | mepindolol.mp. | 26 |
| 28 | carteolol.mp. | 66 |
| 29 | tertatolol.mp. | 11 |
| 30 | bopindolol.mp. | 24 |
| 31 | bupranolol.mp. | 23 |
| 32 | penbutolol.mp. | 61 |
| 33 | cloranolol.mp. | 3 |
| 34 | practolol.mp. | 218 |
| 35 | metoprolol.mp. | 1526 |
| 36 | atenolol.mp. | 1407 |
| 37 | acebutolol.mp. | 242 |
| 38 | betaxolol.mp. | 108 |
| 39 | bevantolol.mp. | 27 |
| 40 | bisoprolol.mp. | 232 |
| 41 | celiprolol.mp. | 97 |
| 42 | esmolol.mp. | 134 |
| 43 | epanolol.mp. | 25 |
| 44 | s-atenolol.mp. | 7 |
| 45 | nebivolol.mp. | 179 |
| 46 | talinolol.mp. | 82 |
| 47 | labetalol.mp. | 441 |
| 48 | carvedilol.mp. | 534 |
| 49 | or/17-48 | 9232 |
| 50 | mortality.mp. or exp mortality/ | 15727 |
| 51 | quality of life.mp. or exp quality of life/ | 7479 |
| 52 | QOL.mp. | 592 |
| 53 | cardiovascular event.mp. | 190 |
| 54 | myocardial infarction.mp. | 6700 |
| 55 | stroke.mp. | 4746 |
| 56 | hospitalization.mp. or exp hospitalization/ | 4328 |
| 57 | hospitalisation.mp. or exp hospitalisation/ | 279 |
| 58 | life expectancy.mp. | 598 |
| 59 | cognitive impairment.mp. | 512 |
| 60 | cognitive status.mp. | 50 |
| 61 | functional status.mp. | 285 |
| 62 | functional impairment.mp. | 185 |
| 63 | renal failure.mp. | 3149 |
| 64 | renal insufficiency.mp. or exp renal insufficiency/ | 1099 |
| 65 | adverse drug event.mp. | 341 |
| 66 | adverse effects.mp. or exp adverse effects/ | 14212 |
| 67 | drug toxicity.mp. or exp drug toxicity/ | 493 |
| 68 | safety.mp. | 32661 |
| 69 | patient safety.mp. or exp patient safety/ | 1784 |
| 70 | falls.mp. | 819 |
| 71 | delirium.mp. or exp delirium/ | 548 |
| 72 | or/50-71 | 78826 |
| 73 | randomized controlled trial.pt. | 0 |
| 74 | controlled clinical trial.pt. | 0 |
| 75 | randomized.ab. | 30879 |
| 76 | placebo.ab. | 23657 |
| 77 | drug therapy.fs. | 0 |
| 78 | randomly.ab. | 8759 |
| 79 | trial.ab. | 22684 |
| 80 | groups.ab. | 34723 |
| 81 | or/73-80 | 79440 |
| 82 | [exp animals/ not humans.sh.] | 0 |
| 83 | 81 not 82 | 79440 |
| 84 | Comparative studies/ | 0 |
| 85 | Follow-up studies/ | 0 |
| 86 | Time factors/ | 0 |
| 87 | chang$.tw. | 51719 |
| 88 | evaluat$.tw. | 107868 |
|  |  |  |
| 89 | reviewed.tw. | 15414 |
| 90 | prospective$.tw. | 13978 |
| 91 | retrospective$.tw. | 13284 |
| 92 | baseline.tw. | 16583 |
| 93 | cohort.tw. | 8657 |
| 94 | case series.tw. | 878 |
| 95 | or/83-94 | 217208 |
| 96 | 13 and 16 and 49 and 72 and 81 and 95 | 72 |

Results = 72

Total results from 4 databases=5424

Duplicates removed = 585

Total = 4839

Limit: 1.1.2011-to 28th September 2016

Total remaining = 903
