# Supplementary material for: Effectiveness and safety of beta blockers in the management of hypertension in older adults: a systematic review to help reduce inappropriate prescribing
Source: BMC Geriatr. 2017 Oct 16;17(Suppl 1):224. doi: 10.1186/s12877-017-0575-4 (PMC5647554; doi:10.1186/s12877-017-0575-4)
Supplement: Supplementary file 2 — Additional evidence for recommendations. (DOCX 19 kb) [file 12877_2017_575_MOESM2_ESM.docx]

**Additional file 2: Additional evidence of interest for recommendations**

| **Source** | **Type of evidence** | **Intervention** |
| --- | --- | --- |
| 1. Kuyper LM, Khan NA. Atenolol vs nonatenolol β-blockers for the treatment of hypertension: a meta-analysis. Can J Cardiol. 2014 May;30(5 Suppl):S47-53. | Meta-analysis | Atenolol vs. non-Atenolol beta blockers |
| 2. De Lima LG, Soares BG, Saconato H, Atallah AN, da Silva EM.Beta-blockers for preventing stroke recurrence. Cochrane Database Syst Rev. 2013 May 31;5:CD007890.doi:10.1002/14651858.CD007890.pub2. Review. Update in: Cochrane Database Syst Rev. 2014;10:CD007890 | Cochrane Review, Meta-analysis | Atenolol vs. placebo |
| 3. Wiysonge CS, Bradley HA, Volmink J, Mayosi BM, Mbewu A, Opie LH. Beta-blockers for hypertension. Cochrane Database Syst Rev. 2012 Aug 15;8:CD002003. Doi:10.1002/14651858.CD002003.pub3. Review. Update in: Cochrane Database Syst Rev. 2012;11:CD002003 | Cochrane Review, Meta-analysis | Any beta blocker vs. placebo or other antihypertensive agents (diuretics, calcium channel blockers, and renin angiotensin system inhibitors) |
| 4. Wiysonge CS, Bradley HA, Volmink J, Mayosi BM, Opie LH. Beta-blockers for hypertension. Cochrane Database of Systematic Reviews 2017, Issue 1. Art. No.: CD002003. DOI: 10.1002/14651858.CD002003.pub5 | Cochrane Review, Meta-analysis | Any beta blocker vs. placebo or other antihypertensive agents (diuretics, calcium channel blockers, and renin angiotensin system inhibitors) |
| 5. Chen JM, Heran BS, Perez MI, Wright JM. Blood pressure lowering efficacy of beta-blockers as second-line therapy for primary hypertension. Cochrane Database. Syst Rev. 2010 Jan 20;(1):CD007185 | Cochrane Review, Meta-analysis | Any beta blocker as second line therapy, mono- vs. combination-therapy |
| 6. Hackam DG, Khan NA, Hemmelgarn BR et al. Canadian Hypertension Education Program. The 2010 Canadian Hypertension Education Program recommendations for the management of hypertension: part 2 - therapy. Can J Cardiol. 2010 May;26(5):249-58. | Evidence based recommendations for the Canadian Hypertension Education Programm | Any beta blocker |
| 7. Leung et al. Hypertension Canada's 2016 Canadian Hypertension Education Program Guidelines for Blood Pressure Measurement, Diagnosis, Assessment of Risk, Prevention, and Treatment of Hypertension. Can J Cardiol. 2016 May;32(5):569-88. | Evidence based recommendations for the Canadian Hypertension Education Programm | Any beta blocker |
| 8. Duodecim Medical Publications Ltd. EBM Guidelines [Internet]: ebm00759 Cerebral Infarction (ischaemic stroke). Helsinki [updated 2013 May17;cited 2016 June 15]. Available from http://www.ebm-guidelines.com | Evidence based guideline | Any beta blocker |
| 9. Dahlof B, Devereux RB, Kjeldsen SE, Julius S, Beevers G, de Faire U, Fyhrquist F, Ibsen H, Kristiannson K, Lederballe-Pederson O, et al([5](#_ENREF_5)). Cardiovascuar morbidity and mortality in the Losartan Intervention for Endpoint reduction in hypertension study (LIFE): a randomised trial against atenolol. Lancet 2002;359:995–1003 | Randomized controlled trial | Losartan vs. Atenolol |
