# Supplementary material for: Effectiveness and safety of beta blockers in the management of hypertension in older adults: a systematic review to help reduce inappropriate prescribing
Source: BMC Geriatr. 2017 Oct 16;17(Suppl 1):224. doi: 10.1186/s12877-017-0575-4 (PMC5647554; doi:10.1186/s12877-017-0575-4)
Supplement: Supplementary file 4 — Risk of bias – Clinical trials. (DOCX 71 kb) [file 12877_2017_575_MOESM4_ESM.docx]

**Additional file 4: Risk of bias - Clinical trials**

The quality appraisal comprises five original randomized controlled trials (intervention studies) [35, 37, 40, 41, 47]. The COPE, Matsuzaki, and MRC main trials are treated as single studies. The secondary analyses studies based on the COPE and MRC trial [36, 38, 39, 41, 48] are not considered in the risk of bias summary. In Ruwald et al. [40] we did use the study protocol [57] when assessing risk of bias.

**Allocation**: **random sequence generation and allocation concealment**

Four of five studies used an adequate random sequence generation and assured concealment of allocation [34, 37, 40, 47].

**Blinding**

Blinding of participants and personnel was assured in one of the included studies [40]. The outcome assessors were blinded in three studies [35, 40, 47]. The absence of blinding might affect the results.

**Incomplete outcome data addressed**

In one study the level of drop-outs and the difference lost to follow-up between intervention and control groups was high [35]. One study provided insufficient information to rate attrition bias as high or low [47].

**Selective reporting**

Three studies [35, 37, 40] were judged to have a low risk of selective reporting bias and one study [34] was rated as high risk.

**Other potential sources of bias**

As mentioned in the introduction five studies were based on secondary analysis of randomized controlled trials [36, 38, 39, 41, 42].Secondary analyses may produce a risk of multiple testing and post hoc definition of endpoints. The results of these studies should only be used for generating hypotheses. Baseline data were reported adequately in all included studies. In most studies physicians, who implemented intervention into practice, were probably involved with patients in both intervention and control group. Therefore the risk of contamination was judged to be unclear. In two studies an appropriate power calculation was performed [37, 47]. Two studies did not achieve the calculated sample size [34, 35] and it remains unclear if this had an impact on the results. One study did not perform a power calculation [36] Intention-to-treat analysis was conducted in two studies [34, 37]. One study provided information on a modified intention to treat analysis [47]. In two studies, insufficient information was provided by the study authors to permit judgment whether an intention-to-treat analysis was performed or not [35, 36]. In two studies a high crossover rate between groups was registered [34, 37].
